# Non-exhaustive DNA methylation-mediated transposon silencing in the black truffle genome, a complex fungal genome with massive repeat element content

**DOI:** 10.1186/s13059-014-0411-5

**Published:** 2014-07-31

**Authors:** Barbara Montanini, Pao-Yang Chen, Marco Morselli, Artur Jaroszewicz, David Lopez, Francis Martin, Simone Ottonello, Matteo Pellegrini

**Affiliations:** Laboratory of Functional Genomics and Protein Engineering, Biochemistry and Molecular Biology Unit, Department of Life Sciences, University of Parma, Parma, 43124 Italy; Institute of Plant and Microbial Biology, Academia Sinica, Taipei, 11529 Taiwan; Department of Molecular, Cell, and Developmental Biology, University of California, Los Angeles, CA 90095 USA; Ecogenomics of Interactions’ Lab, UMR “Tree-Microbe Interactions” INRA-Nancy, Champenoux, 54180 France; Present address: Department of Molecular, Cell, and Developmental Biology, University of California, Los Angeles, CA 90095 USA

## Abstract

**Background:**

We investigated how an extremely transposon element (TE)-rich organism such as the plant-symbiotic ascomycete truffle *Tuber melanosporum* exploits DNA methylation to cope with the more than 45,000 repeated elements that populate its genome.

**Results:**

Whole-genome bisulfite sequencing performed on different developmental stages reveals a high fraction of methylated cytosines with a strong preference for CpG sites. The methylation pattern is highly similar among samples and selectively targets TEs rather than genes. A marked trend toward hypomethylation is observed for TEs located within a 1 kb distance from expressed genes, rather than segregated in TE-rich regions of the genome. Approximately 300 hypomethylated or unmethylated TEs are transcriptionally active, with higher expression levels in free-living mycelium compared to fruitbody. Indeed, multiple TE-enriched, copy number variant regions bearing a significant fraction of hypomethylated and expressed TEs are found almost exclusively in free-living mycelium. A reduction of DNA methylation, restricted to non-CpG sites and accompanied by an increase in TE expression, is observed upon treatment of free-living mycelia with 5-azacytidine.

**Conclusions:**

Evidence derived from analysis of the *T. melanosporum* methylome indicates that a non-exhaustive, partly reversible, methylation process operates in truffles. This allows for the existence of hypomethylated, transcriptionally active TEs that are associated with copy number variant regions of the genome. Non-exhaustive TE methylation may reflect a role of active TEs in promoting genome plasticity and the ability to adapt to sudden environmental changes.

**Electronic supplementary material:**

The online version of this article (doi:10.1186/s13059-014-0411-5) contains supplementary material, which is available to authorized users.

## Background

DNA methylation is a heritable epigenetic modification involved in the regulation of a variety of processes ranging from development to genomic imprinting, gene regulation and transposon silencing [1]. It is found in animals, plants, and some fungi, but has been lost in various invertebrate lineages [2].

A genome-wide picture of DNA methylation has emerged recently from methylome studies carried out on more than 20 eukaryotic organisms belonging to four different lineages [3–7]. As revealed by these studies, DNA cytosine methylation occurs either within symmetric CG/CHG, or asymmetric CHH sequence contexts (where H stands for A, C, or T). The latter are frequently observed in plants and fungi, whereas symmetric, especially CG methylation, predominates in vertebrate genomes (reviewed by [8]). Promoter and gene-body methylation marks are common in higher eukaryotes, where they provide an additional level of gene regulation (both negative and positive), while inactivation of transposons and other repeated elements appears to be the main purpose of DNA methylation in fungi (reviewed by [2]). DNA methylation is an essential process in both plants and animals; its disruption leads to a variety of abnormal phenotypes, including altered development and cancer, but it appears to be dispensable in the model filamentous fungus *Neurospora crassa* and in various invertebrates [2,9,10].

The multiplicity and sequence specificity of DNA methyltransferases (DMTs) is also beginning to be delineated (reviewed by [2,8]). The presence of two particular DMTs (Dnmt1 and Dnmt3) is associated with CG methylation in vertebrates and in certain plants, whereas a third DMT, the chromomethylase CMT, is associated with the non-CG methylation found in four of the six plant genomes examined so far. In filamentous fungi, the number of predicted DMTs (Dnmt1 or Dnmt1-related) ranges from one to two, but their sequence specificity and mode of action are not yet fully understood. Additional putative methyltransferases are present in some ascomycetes belonging to the Pezizomycotina (for example, the RID and Masc1 proteins of *N. crassa* and *Ascobolus immersus*), which are responsible for premeioitic genome defense processes such as “repeat-induced point mutation” (RIP) and “methylation-induced premeiotically” (MIP), respectively [11,12].

*Neurospora*, with an estimated 2% bulk methylation level and a Dnmt1-related, functionally active methyltransferase (DIM-2), is the best-studied fungus with regard to DNA methylation. The molecular mechanisms leading to 5-methylcytosine (5mC) formation that were first discovered in this fungus seem to be quite conserved and paved the way to a deeper understanding of DNA methylation in other organisms [13]. Nevertheless, a number of DNA methylation variations and idiosyncrasies appear to be present in fungi [14]. For example, fungi seem to lack RNA-dependent methylation processes [15]. Moreover, DNA methylation is not present in a variety of unicellular yeasts such as *Saccharomyces cerevisiae* and *Schizosaccharomyces pombe*, and also in some filamentous ascomycetes evolutionarily related to *Neurospora* such as *Aspergillus nidulans* and *Aspergillus flavus* [16,17]. Another unusual situation occurs in the dimorphic yeast *Candida albicans*, where methylation has been shown to target and modulate the transcription of genes [18].

In *Neurospora*, methylated DNA is mainly found in transposable element (TE) relics produced by RIP [10], which relies on cytosine methylation coupled to 5mC deamination of CA dinucleotides brought about by the putative methyltransferase RID, leading to the accumulation of C:G to T:A transitions in repeated elements [12]. MIP is a silencing mechanism that also relies on DNA cytosine methylation, but is not directly coupled to 5mC deamination. It has been first documented in the ascomycete *A. immersus*, where it is carried out by the RID homolog Masc1, and causes the repression of methylated repeated elements as well as the suppression of homologous recombination [11]. In both *N. crassa* and *A. immersus*, DNA methylation could be reverted to varying extents by treatment with 5-azacytidine (5-aza) and other cytosine analogues [19–21]. Curiously, even in filamentous fungi lacking DNA methylation, such as *Aspergillus* spp., 5-aza has been shown to induce a mycelial phenotype modification, characterized by overgrowing and dispersed hyphae, designated as “fluffy” ([22,23] and references therein).

A general trend toward CG-targeted methylation of transposable or otherwise repetitive elements has been documented by methylome analyses of five fungi representative of the ascomycetes, basidiomycetes and zygomycetes [7]. Despite an estimated divergence time of more than 1 billion years between the three sampled basidiomycetes and the zygomycete *Phycomyces blakesleeanus*, the methylation patterns of the four fungi were overall very similar, with a marked preference for CG target sequences located within TEs or other repeated elements, and the virtual absence of gene methylation. In contrast, the methylome of *Uncinocarpus reesii*, an ascomycete distantly related to *N. crassa* that contains a DMT homologous to DIM-2 plus a RID/Masc1 homolog, revealed rather unexpected features, such as a marked preference for CG rather than CA dinucleotides predicted as RIP sites, a reduced CG methylation and appreciable levels of gene-body methylation specifically targeting exon sequences as in plants [7]. The divergent patterns observed in *U. reesii* point to the diversity of DNA methylation mechanisms in fungi and to the need for in-depth investigations across multiple closely related species, especially when they are characterized by distinctively different lifestyles and unusual genome structures.

With this goal in mind, we have determined the genome-wide DNA methylation profiles associated with the different lifecycle stages of the black truffle *Tuber melanosporum*, a macrofungus and a highly appreciated gastronomic delicacy produced by an ectomycorrhizal ascomycete symbiont found throughout southern Europe. *T. melanosporum* features one of the largest genomes (125 Mb), with an exceptionally high TE and repetitive DNA content (>58%), among the fungi that have been sequenced so far [24]. As obligate outcrossing organisms, truffles are bound to a sexual mode of propagation, which together with TEs has been proposed to be a major force driving the evolution of DNA methylation [2,25]. *T. melanosporum* belongs to the Pezizales, a largely unexplored group of ascomycetes that includes *A. immersus*, a fungus relying on premeiotic DNA methylation as a means to control repetitive element proliferation [11,26].

Our methylome analysis revealed an extensive DNA methylation that selectively targets TEs, covers about 44% of the cytosines in the truffle genome, and could be partly reverted by 5-aza treatment with a concomitant increase of TE transcription. The methylation pattern in *Tuber* shares a number of features with *Ascobolus* MIP, but it is less exhaustive, with approximately 300 unmethylated or poorly methylated TEs (especially small-sized elements) that escape modification and remain transcriptionally active. Indirect evidence in favor of a residual TE activity and its potential contribution to genome plasticity was provided by the discovery of amplified genomic regions enriched in transcriptionally active transposons leading to extensive copy number variation (approximately 7% of the reference genome size).

## Results

We generated genome-wide DNA methylation profiles at single-base resolution of the saprobic free-living mycelium (FLM), truffle fruitbody (FB) and ectomycorrhizal (ECM) developmental stages of *T. melanosporum* (using bisulfite sequencing (BS-seq)). We aligned bisulfite-converted reads generated by an Illumina sequencer against the truffle genome [24] using the BS Seeker program [27]. We achieved 32X, 35X and 1.2X coverage per strand for FB, FLM and ECM, respectively (Table S1 in Additional file 1). The ECM sample contained substantial amounts of root cells from the hazelnut (*Corylus avellana*) host tree, whose genome has not yet been determined. Even at this low coverage (1.2X), we were able to delineate global, low-resolution methylation profiles for the symbiotic ECM stage of the *Tuber* lifecycle.

Methylation levels were estimated for each cytosine covered by at least four reads (see Materials and methods for details). The results presented below are centered on the highly covered FB and FLM methylomes; most of the corresponding ECM data are separately presented in Tables S1 and S2, Figures S1 and S3-S7, in Additional file 1.

### Genome-wide DNA methylation profiles associated with the different developmental stages of *T. melanosporum*

**Figure 1:**
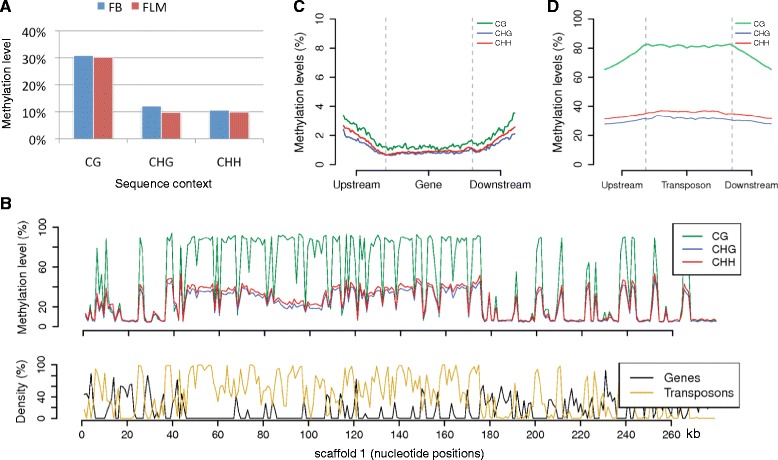
**Global patterns of DNA methylation in** ***T. melanosporum.*** **(A)** Average methylation levels in fruitbody (FB) and free-living mycelium (FLM). **(B)** Methylation levels of CG, CHG, and CHH sites in scaffold 1 (FB); density of genes and transposons in the same scaffold is shown in the bottom track. **(C,D)** Meta-plots of FB methylation levels in genes (C) and transposable elements (D).

The data on common sites of FB and FLM samples cover more than 90% of the cytosines in the truffle genome. We found that 44% and 41% of these covered cytosines are methylated in FB and FLM, respectively. On average, the methylation level is approximately 30% for CG sites and 10% for non-CG sites (Figure 1A; Table S2 in Additional file 1). Similar to previous reports in plants and mammals, CG methylation levels in truffle display a bimodal distribution, with CG sites either highly (>80%) or weakly (<10%) methylated. Most non-CG sites have lower levels of methylation (<50% on average) (Figure S1 in Additional file 1). FB and FLM have very similar bulk methylation levels, although FB is slightly more methylated (∆ methylation levels = +0.6, +2.3, and +0.8% for CG, CHG, and CHH sites, respectively; Table S2 and Figure S2 in Additional file 1).

We used 260,000 common cytosines (58K CG, 54K CHG, and 147K CHH sites), sufficiently covered in all three samples, for the comparison with ECM. Despite the lower methylation levels of the common sites utilized for this comparison, we found a relative distribution (and a preference for CG sites) similar to that observed in FB and FLM. The average methylation level for ECM is about 8%, 5% and 4% for CG, CHG and CHH sites, respectively (Table S2 in Additional file 1). Average global methylation levels are highest in FB, followed by ECM, and lowest in FLM, although they differ by no more than 2% from each other. It should be noted, however, that due to the generally lower mapability of ECM reads, weakly methylated gene-rich regions are likely to be over-represented.

A large-scale view of FB methylation levels measured in different sequence contexts (that is, CG, CHG, and CHH) across the first *T. melanosporum* genome scaffold is shown in Figure 1B. Similar plots for FLM and ECM are reported in Figure S3 in Additional file 1, and genome-wide views for the three samples are shown in Figure S4 in Additional file 1. Methylation profiles exhibit a mosaic pattern [28], with DNA methylation concentrated in TE-rich regions and generally depleted in genes (Figure 1B). As further shown in the meta-plots (Figure 1C,D; Figure S5 in Additional file 1), methylation profiles determined across genes and TEs in FB confirm that genes and exons are indeed very weakly methylated (approximately 1%), whereas TEs are highly methylated at both CG (approximately 80%) and non-CG (approximately 30%) sites. Despite the strikingly different methylation levels associated with genes and TEs, the sequence motifs for CG and non-CG methylation and their relative preference turned out to be essentially the same when determined on total or transposon-associated DNA (Figures S6 and S7 in Additional file 1). Although the rDNA cluster is not fully assembled in the present version of the *T. melanosporum* genome, multiple rRNA genes were identified on scaffolds 298 and 355 and they were found to display a high (70 to 90%) level of DNA methylation.

These results indicate a strong and preferential TE methylation in all three developmental stages of *T. melanosporum* and suggest that DNA methylation is likely involved in the control of transposon activity.

### DNA methylation is negatively associated with transposon expression, but not gene expression

We next examined the relationship between DNA methylation and expression using an RNA-seq dataset generated from the same biological samples (FLM and FB) used for the methylation analysis. Since gene methylation is generally low, we expected a weak association with gene expression. Not surprisingly, we found essentially no correlation between gene (promoters and gene bodies) methylation and gene expression (Figure S8 in Additional file 1).

**Figure 2:**
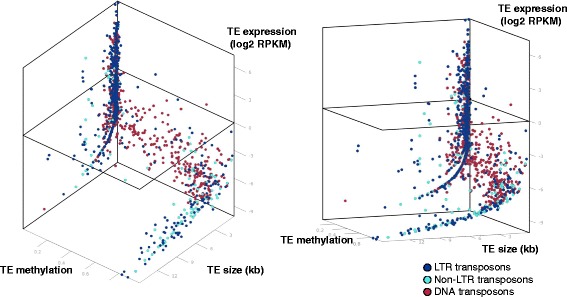
**Transposon methylation and expression.** CG methylation levels of individual TEs plotted against their size (kb) and expression levels (Log2 RPKM (reads per kilobase per million mapped reads)) in FB. A three-dimensional scatter plot is shown from two views. TEs belonging to different classes are displayed as different color dots as indicated. Only TEs with detectable expression levels (RPKM >0) are shown; a total of 1,414 TEs for FB. The plane cut at log2 RPKM = 0 (corresponding to RPKM = 1) was set as a threshold for expressed TEs. The results of a similar analysis conducted on FLM and on TEs with undetectable expression levels are shown in Figure S9 in Additional file 1.

In contrast, we found a significant negative correlation between TE expression and TE methylation levels in both FB and FLM (Figure 2; Figure S9 in Additional file 1), where highly expressed TEs were found to be significantly less methylated than lowly expressed TEs. In accordance with previous reports indicating that methylation acts as a TE silencing mechanism [29,30], these results suggest that TE methylation levels in *Tuber* are associated with transcriptional repression of TEs.

**Figure 3:**
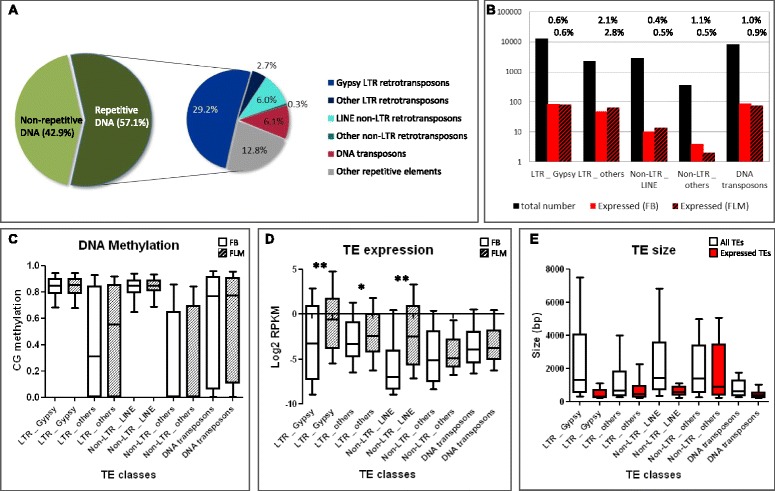
**DNA methylation and expression levels in different TE subclasses. (A)** Pie-chart diagram of TE distribution and relative occupancy (expressed as cumulative size) in the *T. melanosporum* genome. **(B)** The total number of TEs in each subclass (black bars) and the number of expressed TEs (RPKM >1) in FB (red bars) and FLM (striped red bars) are indicated. Percentage values shown above solid red and striped bars indicate the fraction of expressed TEs in each subclass. **(C,D)** Box plots of CG methylation **(C)** and expression levels **(D)** for the indicated TE subclasses in FB (white bars) and FLM (striped bars). Median methylation levels in **(C)** are close to zero for non-LTR retrotransposons. TE classes with different expression levels in FB and FLM are indicated (*t*-test significance: **P-value <0.001; *P-value <0.05). Log2 RPKM values in **(D)** are only reported for TEs with at least one mapped read (RPKM >0). **(E)** Box plot of all TEs (white bars) and TEs expressed in at least one developmental stage (FB/FLM) (red bars).

TE methylation also correlates with transposon size. As shown in Figure 2 and Figure S9 in Additional file 1, small TEs exhibit a wide-range of CG methylation levels, while larger TEs (>1.5 kb) are more methylated. Furthermore, and in accordance with the above described inverse relationship between TE methylation and expression, large TEs tend to be expressed at very low levels: only 0.23% of those larger than 1.5 kb are expressed at all (that is, above a RPKM (reads per kilobase per million mapped reads) = 1 threshold), whereas 1.64% of TEs smaller than 1.5 kb appear to be expressed. In both FLM and FB, the most highly expressed TEs are small (≤1.5 kb) and lowly methylated (Figure 2; but see also Figure S9 in Additional file 1). We analyzed TE methylation and expression levels in more detail with respect to previously classified groups of *Tuber* TEs [24]. In *T. melanosporum*, nearly half of the classified TEs are class I retroelements belonging to the long terminal repeat (LTR) retrotransposon Ty3/Gypsy superfamily, accounting for about 30% of the genome of this fungus (Figure 3A). The second most abundant class comprises class II DNA transposons (21% of all classified TEs), followed by LINE non-LTR-retrotransposons (10% of all classified TEs), non-Gypsy LTR retrotransposons (8%) and a small fraction of non-LTR, non-LINE retrotransposons (less than 1%) [24]. Additional, unclassified repetitive elements (“Other repetitive elements” in Figure 3A) rarely contain recognizable open reading frames typically associated with TEs (<1%) and were thus excluded from analysis. As shown in Figure 3B, a few transposable elements in each class are expressed (RPKM >1) in at least one developmental stage (78 TEs expressed in FLM, 81 in FB, and 149 TEs expressed in both stages). The fraction of TEs expressed at RPKM >1 ranges from 0.4% for LINE non-LTR retrotransposons in FB to 2.8% for non-Gypsy LTR-retrotransposons in FLM (Figure 3B).

Members of the Gypsy and LINE superfamilies, representing 59% of all classified TEs in *T. melanosporum*, are the most methylated, while non-LTR, non-LINE retrotransposons are the least methylated (Figure 3C). Interestingly, in each class, TE expression levels are generally higher in FLM than in FB (*P* < 0.001 for Gypsy and LINE elements; *P* < 0.05 for non-Gypsy LTR retrotransposons; Figure 3D), and expressed TEs are usually smaller than non-expressed TEs (Figure 3E). The sole exception is represented by non-LTR, non-LINE retrotransposons, for which the six expressed elements exhibit a fairly broad size range (Figure 3D,E).

**Figure 4:**
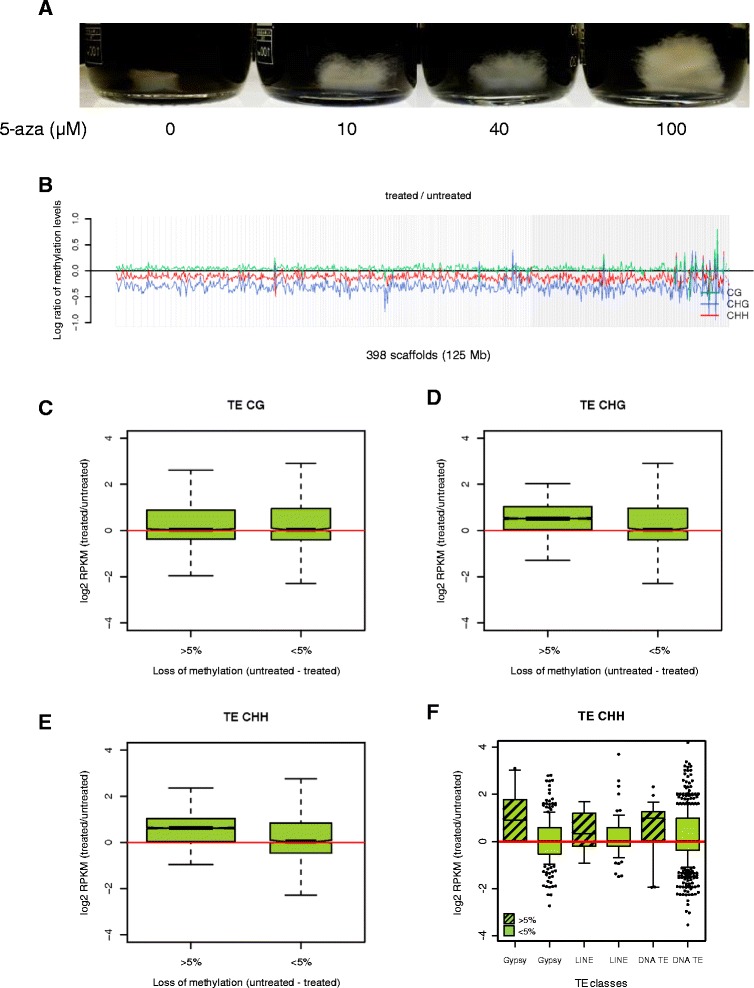
**DNA methylation profiles and TE expression levels of free-living mycelia treated with 5-azacytidine. (A)** Dose-dependent induction of the ‘fluffy‘ phenotype in free-living mycelia treated for 45 days with increasing concentrations of 5-aza. **(B)** Genome-wide comparison of DNA methylation levels in 5-aza (100 μM)-treated versus untreated mycelia. **(C)** Box plot representation of the fold-change in expression levels of TEs with different CG delta methylation levels (5-aza untreated - treated). **(D,E)** Same as (C) for CHG and CHH delta methylation levels as indicated. *t*-Test *P*-values for TE expression changes associated with CHG and CHH delta methylation levels are 0.1436 and 0.01367, respectively. **(F)** Fold-change TE expression levels as a function of CHH methylation levels for the indicated TE subclasses; delta CHH methylation levels (untreated - treated) >5% or <5% are shown as striped and unstriped bars, respectively. Only TE subclasses with at least 10 members are shown.

Further evidence in favor of an inverse relationship between TE methylation and expression was provided by BS-seq and RNA-seq analyses of mycelia treated with the demethylating agent 5-aza. Given the known instability of 5-aza in aqueous solution and the lack of information on the timing of its potential effects and toxicity in *Tuber*, we initially explored different 5-aza concentrations and treatment conditions. As shown in Figure 4A, a dose-dependent transition to a fluffy-like phenotype, without any apparent toxicity, was observed in *T. melanosporum* mycelia cultured for 45 days in the presence of different 5-aza concentrations. This change was maximal at 100 μM and mycelia treated with this 5-aza concentration, along with control untreated mycelia, were used as sources of genomic DNA and RNA for BS-seq and RNA-seq. As revealed by this analysis (Figure 4B), a decrease of global and TE-associated methylation levels (see also Tables S3 and S4 in Additional file 1) was detected in 5-aza-treated mycelia. Importantly, TE demethylation primarily affected symmetric (CHG) and asymmetric (CHH) non-CG sites and was accompanied by an increase of TE expression (Figure 4C-F).

### The association between TE methylation and nearby gene expression

**Figure 5:**
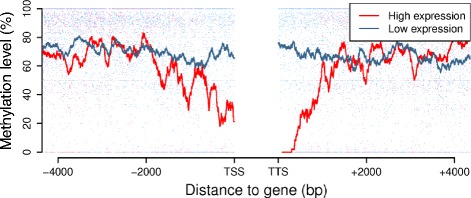
**TE methylation in the proximity of genes with different expression levels.** CG methylation levels of TEs located upstream or downstream of highly expressed genes (top 25%; red dots) and lowly expressed genes (bottom 25%; blue dots). Red (highly expressed genes) and blue (lowly expressed genes) lines represent the moving average of methylation levels in 100 bp windows. *t*-Test *P*-values of the differences in percentage TE methylation between highly and lowly expressed genes were 2.2 × 10^-16^ for TE-gene distances ≤0.5 kb and ≤10^-5^ for TE-gene distances between 0.5 and 1 kb.

In *Arabidopsis thaliana*, a repressive effect of DNA methylation-mediated TE silencing on the expression of nearby genes has been reported, resulting in the preferential loss of methylated TEs from gene-rich chromosomal regions [29]. To investigate whether a similar phenomenon occurs in truffle, we determined the methylation levels of TEs located at a relatively close distance (upstream or downstream) from genes. As shown in Figure 5, we found that the cytosines of TEs close to highly expressed genes (up to 1 kb, upstream or downstream of the transcription start or termination sites) tend to be less methylated than those located near lowly expressed genes and that this difference increases with increasing proximity to the genes (*t*-test *P*-value of 2.2 × 10^-16^ for TE-gene distances ≤0.5 kb). The same pattern is observed in both FB and FLM, in CG as well as non-CG sequence contexts (Figure S10 in Additional file 1), whereas no difference with respect to DNA methylation was observed for non-TE regions close to highly or lowly expressed genes (Figure S11 in Additional file 1).

We also asked whether gene-neighboring TEs (that is, TEs preceded and/or followed by a gene within a 1 kb distance) are more likely to be expressed. In the truffle genome only 2.2% of TEs are located at a distance less than 1 kb from coding genes. This likely reflects the structural organization of the *Tuber* genome, which is made of small blocks of genes interspersed within large TE islands [24]. Despite the low frequency of gene-proximal TEs, we found that these TEs are preferentially located near genes with high to median expression levels. Also, in accordance with the observation that cytosine residues of TEs close to highly expressed genes tend to be less methylated and that expressed TEs exhibit a clear trend toward hypomethylation, we found that about 14% of expressed TEs are close to genes (an over six-fold enrichment with respect to the total fraction of gene-proximal TEs).

### DNA methylation and genome plasticity

**Figure 6:**
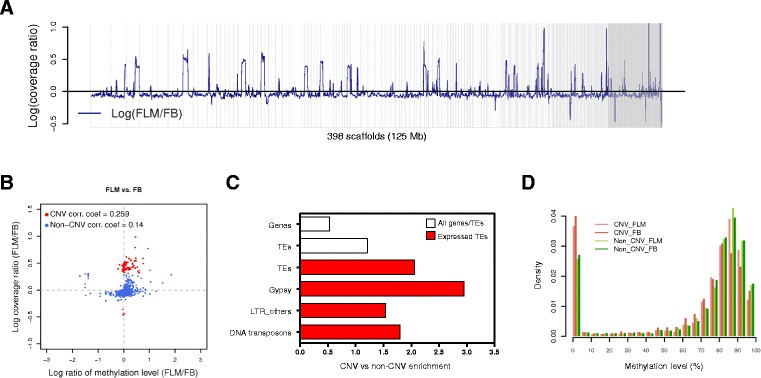
**Copy number variation analysis. (A)** Log (ln) ratio of read coverage between the FLM and the FB genomes. **(B)** Scatter plot of copy number variation versus methylation differences between FB and FLM; the log ratio of methylation levels (FLM/FB) and the log (ln) ratio of coverage are on the x-axis and y-axis, respectively. **(C)** Fold-enrichment of genes and TEs and associated expression features in CNV versus non-CNV regions. **(D)** Distribution of methylation levels in CNV and non-CNV regions.

The coverage of BS-seq data across the genome also revealed copy number variation (CNV) between FLM and FB. Specifically, we identified 107 genomic regions with significant CNV (defined as a 100 kb window with a |Log coverage ratio (FLM/FB)| ≥ 0.3), corresponding to 7.3% of the genome, 102 (95%) of which were independently confirmed by standard Illumina sequencing performed on non-bisulfite-treated FLM genomic DNA (Table S1 in Additional file 1). Most of these regions (96%) are amplified in FLM with respect to FB (Figure 6A). Interestingly, CNV regions weakly correlate with methylation fold change; that is, they are, on average, more methylated in the genome (and developmental stage) in which they are amplified (Figure 6B).

CNV regions are characterized by a 20% increase in TEs and a 50% depletion of genes compared to the rest (non-CNV portion) of the genome (Figure 6C). When the analysis is restricted to TEs only, a comparison of the distributions of transposon methylation in non-CNV versus CNV regions shows that TEs are significantly less methylated in CNV than in non-CNV regions (Kolmogorov-Smirnov test, *P* < 10^-5^; Figure 6D) and that these TEs are also more likely to be expressed (Figure 6C). For example, the approximately 7% of genomic DNA associated with CNV regions comprises approximately 9% of the TEs present in the reference genome and this fraction rises to approximately 14% if one specifically considers expressed TEs. The latter group is particularly enriched in expressed Gypsy elements, whose members located in CNV regions account for approximately 19% of the entire subset of expressed Gypsy retrotransposons in the *T. melanosporum* genome (Figure 6C).

Collectively, these data further indicate that DNA methylation and TE repression are less extensive in FLM than in FBs, and even more so in CNV regions of the genome.

### DNA methyltransferases

Two putative DNA methyltransferases, TmelDnmt1 and TmelDnmt2, are encoded by the truffle genome [24]. A similar DMT multiplicity is common in filamentous fungi regardless of their evolutionary relatedness [2,15]. Notable exceptions are the presence of only one RID/Masc1-like gene in some aspergilli lacking DNA methylation [16,17] and the postulated existence of a third DMT in *A. immersus* [11]. The latter is confirmed by a homology search conducted on the recently sequenced genome of *A. immersus* (accessible at the US Department of Energy Joint Genome Institute) that revealed the presence of an additional putative DMT designated as Masc3.

**Figure 7:**
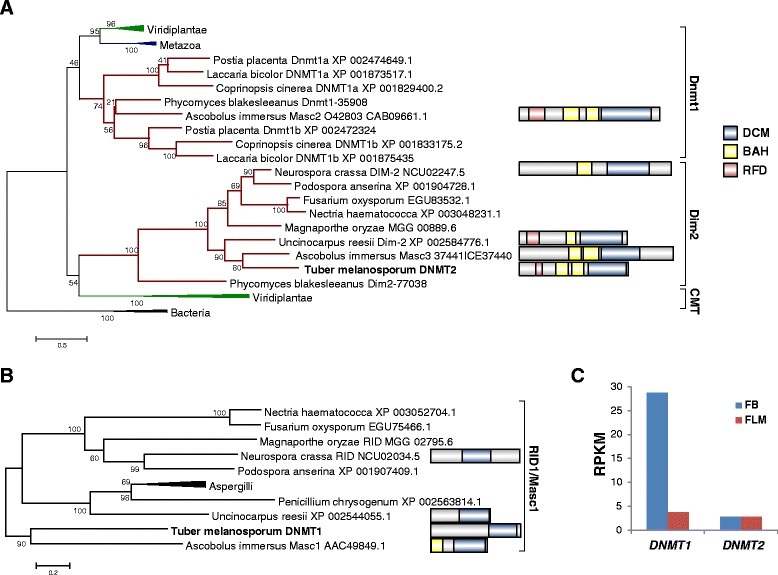
**Phylogeny and expression levels of putative** ***T. melanosporum*** **DMTs. (A)** Maximum likelihood tree based on the catalytic domains of DNMT1 and DNMT1-related DMTs. Bacterial DMTs were used as outgroup; divergent enzymes belonging to the DNMT3 group were not considered. **(B)** Maximum likelihood analysis conducted as in (A) on RID-like proteins from various fungi. Source organism name and accession numbers are indicated for each sequence. Bootstrap values (1,000 replicates) are indicated for each branch. Additional DNMT1 domains, besides the catalytic domain (DCM), are indicated on the right side near to ‘reference’ fungal proteins (BAH, bromo-adjacent homology; RFD, replication foci domain). **(C)** Expression levels (RPKM) of the RID/Masc1-like (TmelDNMT1, GSTUMT00012206001; left bars) and the DIM-2-like (TmelDNMT2, GSTUMT00003328001; right bars) *Tuber* DMTs in FB (blue bars) and FLM (red bars).

To gain insight into the putative methyltransferases of *T. melanosporum*, we first aligned their predicted catalytic domains (DCM) with those of a large set of fungal methyltransferases, including a few functionally validated DMTs. Based on specific motif signatures, sequence similarity to known non-fungal DMTs and/or origin from a RIP- or MIP-proficient fungus (*N. crassa* and *A. immersus*, respectively), we assigned these DMTs to either the DIM-2/DNMT1 or the RID/Masc1 classes. Following this initial classification, we used the above methyltransferases and known bacterial, plant, and metazoan DMTs [7] for a maximum likelihood phylogenetic analysis aimed at identifying the orthologs of the two putative *Tuber* DMTs. As shown in Figure 7, which also illustrates the predicted domain composition of a specific subset of fungal methyltransferases, the orthologs of TmelDnmt1 and TmelDnmt2 are *Ascobolus* Masc1 and Masc3, respectively.

Clustering of fungal DIM-2 with Viridiplantae CMT methyltransferases as well as clustering of fungal, Viridiplantae and Metazoa DNMT1 methyltransferases is apparent in Figure 7A and resembles a previously reported DMT tree topology [2,7].

Both *Tuber* DMTs are expressed at above background levels in FLM and FB, with a stronger expression of *TmelDnmt1* in the latter developmental stage (Figure 7C). Also present in *Tuber* are expressed homologs of all the components (for example, the histone methyltransferase DIM-5, the H3K9me3-binding protein HP1, the H3S10p phosphatase PP1, and the DNA methylation modifier DMM-1) that assist DNA methylation in *Neurospora* [13] (Figure S12 in Additional file 1).

While *U. reesii*, whose putative DIM-2 DMT belongs to the same cluster as TmelDnmt2 (Figure 7B), is thought to be a RIP-proficient fungus (albeit with a different target site preference compared to *N. crassa* [7]), *A. immersus* is a member of the same group of filamentous ascomycetes (Pezizales) to which *Tuber* belongs [26] and the fungus in which MIP was first discovered [11].

Previous *in silico* analyses based on RIPCAL [24,31] and on dinucleotide frequency distribution analysis [32] found no evidence for RIP in *T. melanosporum*. We obtained a similar result from a cross-species comparison using the composite RIP index (CRI) [33] (see Materials and methods) to measure CA > TA enrichment within repeated sequences from *Tuber* and other organisms’ genomes. As shown in Figure S13A in Additional file 1, *N. crassa* has the highest score for CA to TA transitions and the lowest score for CG to TG mutations, whereas the opposite situation (Figure S13B in Additional file 1) is observed in *U. reesii*, a filamentous ascomycete predicted to be RIP’d at CG dinucleotides [7]. Compared to these two reference fungi, *T. melanosporum* did not show any significant enrichment in either mutation, with score distributions similar to those determined for RIP-lacking organisms such as the yeast *S. cerevisiae*.

## Discussion

The goal of this work was to determine how an extremely TE-rich organism such as *T. melanosporum* exploits DNA methylation to cope with the more than 45,000 repeated elements that populate its genome. In *Tuber*, DNA methylation targets TEs in a nearly exclusive manner. The functional consequence of this preferential targeting is that TE, but not gene, expression is largely repressed and that the extent of such repression is proportional to methylation levels. A significant difference compared to the other fungi that have been examined at the methylome level so far [7] is the extent of DNA methylation, which in the truffle genome covers more than 44% of cytosine residues, with a marked preference for CpG dinucleotides. This is considerably higher than the methylation levels estimated in other fungi and sets *Tuber* in the top rank of methylation-proficient organisms, including plants [2,8,34].

Two TE silencing mechanisms, RIP and MIP, both of which rely on DNA methylation albeit with some major differences, have been documented in filamentous fungi. The hallmark of RIP is irreversible, repeated sequence mutation via methylation coupled to deamination of C residues within the CpA dinucleotide context [12]. In MIP, instead, which has so far been described only in the pezizalean ascomycete *A. immersus*, repeated sequence silencing primarily relies on heritable cytosine methylation, which may be followed by, but is not strictly coupled to, 5mC deamination [11]. Therefore, while RIP is a predominantly irreversible genetic mechanism, MIP more closely resembles reversible epigenetic control as in higher eukaryotes.

No evidence for RIP in *Tuber* was provided by *in silico* analyses used to estimate the TpA/ApT ratio (the expected consequence of methylation-coupled 5mC deamination) throughout the truffle genome (Figure S13A,B in Additional file 1) [24,32]. We also compared the sequence context surrounding methylated and unmethylated cytosines in the *Tuber* genome and found that methylated, but not unmethylated, sites (especially CG sites) are enriched in TA dinucleotides (Figures S6 and S7 in Additional file 1). In principle, this might be viewed as evidence for RIP, where RIP’d sites (bearing CA to TA transitions) have been shown to act as signals for the subsequent methylation of nearby cytosines regardless of their sequence context [15]. It should be noted, however, that in *Neurospora*, multiple and fairly extensive A:T-rich sequences (for example, [TAAA]n or [TTAA]n), rather than relatively short 5mC-flanking T/A sequences as we find in *Tuber*, have been shown to represent the strongest signals for methylation [35]. Furthermore, G:C base pairs adjacent to ApT dinucleotides, as in the *Tuber* sequence context, tend to suppress DNA methylation in *Neurospora* [13,35]. An alternative possibility is that A:T enrichment close to methylated cytosines reflects spontaneous deamination of heavily methylated (MIP’d) regions, as observed in the genomes of higher-eukaryotic non-RIPping organisms, including humans [28,36], rather than a RIP signature. Indeed, both in FB and FLM, SNP density, and specifically C > T transitions, is much higher in highly methylated (CG methylation >40%) compared to hypomethylated (CG methylation <40%) transposons (Figure S14 in Additional file 1). A further potential cause of the increased mutation rate (SNP density) observed in sequences neighboring highly methylated TE regions, including the *Tuber* genome, may be error-prone repair of gaps generated near to deaminated 5-methyl cytosines [37].

Additional distinguishing features of MIP and RIP, whose evolutionary consequences are expected to be very different, include: i) the marked preference for symmetric CpG methylation sites observed in *Ascobolus* MIP’d regions, compared to the more ubiquitous and less sequence context-dependent cytosine methylation observed in *Neurospora*; ii) the more coextensive nature of MIP-associated DNA methylation compared to RIP methylation, which, by taking place after initial RIPping and being actually signaled by RIP’d sequences, tends to extend beyond the borders of the duplicated regions; and iii) a more prevalent role of so-called “maintenance methylation” in the propagation of MIP-derived DNA methylation, as opposed to the recurrent *de novo* methylation events triggered by RIP mutations [11].

Also with regard to these specific differential features, our methylome data strongly favor MIP as the process responsible for TE silencing in *T. melanosporum*. In fact, we observed a marked (approximately 3:1) bias for CpG versus non-CpG sites, and TE methylation was found to be largely coextensive with repeat size. In fact, TEs exhibited highly methylated profiles compared to their flanking regions (Figure 1D), whereas the opposite situation was apparent in the case of genes, especially highly expressed genes, where TE methylation was found to be strongly reduced near to transcription initiation and termination sites (Figures 1C and 5; Figures S5 and S10 in Additional file 1). TE-restricted methylation resembling the so-called ‘mosaic methylation’ typical of invertebrate, but also plant (especially *Arabidopsis*), genomes could be instrumental in preventing spreading of potentially repressive effects associated with TE methylation, particularly in the case of highly expressed genes. Specific *trans*-acting components such as the *Neurospora* DNA methylation modulator-1 (DMM-1) protein [19], an expressed homolog of which is present in the *T. melanosporum* genome (Figure S12 in Additional file 1), can also prevent spreading of DNA and histone methylation from inactivated TEs to nearby genes. Another important safeguard element, in this regard, may be represented by the very structure of the *Tuber* genome, where relatively small blocks of genes are separated by large TE-containing, intergenic regions, with relatively few cases of genes nested within TE-rich regions and vice versa [24]. Indeed, less than 5% of TEs are found within a 2 kb distance from genes in the *T. melanosporum* genome, with a preference for genes expressed at high or median levels. This fraction increases by about four-fold if only expressed TEs are taken into account, suggesting that the predominantly unmethylated state of genes, especially highly expressed genes, can spread into (or otherwise influence the methylation state of) neighboring TEs.

The third point bears on ‘maintenance methylation’, which in the case of MIP implies the faithful and long-term perpetuation of the methylation state first established in the premeiotic stage. As suggested by the high overall similarity of the methylation profiles determined in a field-collected FB and in vegetative FLM propagated *in vitro* for multiple generations, a rather robust maintenance capacity appears to be another distinguishing feature of DNA methylation in *Tuber*.

Sequence repeats with an identity ≥80% and a size ranging from a minimum of approximately 400 bp to approximately 600 bp depending on their tandem or ectopic arrangement, respectively, are targeted by both MIP and RIP [11]. A similar, but somewhat broader size range for TE methylation was revealed by the *Tuber* methylome, where varying methylation levels are associated with small TEs, whereas TEs larger than 1.5 kb are consistently more methylated. Importantly, TE expression appears to be inversely related to methylation levels, yet about 300 unmethylated or lowly methylated TEs, especially small-sized elements, were found to be transcriptionally active, with a trend toward higher expression levels in free-living mycelium compared to FBs (Figures 2 and 3). Conversely, a higher DNA methylation level, at both CpG and non-CpG sites, was detected in FBs (Figure S2 in Additional file 1).

Ascospores represent a tiny fraction of *Tuber* FBs, which are mainly composed of maternal haploid cells forming the so-called gleba [38]. Since we used total FB tissue for methylome analysis, it is unlikely that the higher DNA methylation and the generally lower TE expression levels observed in FB are due to a more stringent TE control during the sexual stage of the *Tuber* lifecycle. A more plausible explanation is that the different methylation levels detected in FB and FLM reflect the different origins of the two samples. In fact, while the FB tissue was derived from a natural FB immediately frozen after harvest, genomic DNA for FLM methylome analysis was extracted from a *T. melanosporum* vegetative mycelium (strain Mel28 [24]) cultured *in vitro* for more than 20 years. Therefore, despite the overall consistency of DNA methylation maintenance suggested by the high global similarity of the FB and FLM methylomes, it is not too surprising that some methylation loss, and concomitant TE activation, may have occurred during such an extended period of *in vitro* culture. Such a scenario, which is consistent with the decreased methylation levels brought about by 5-aza treatment, is reminiscent of the situation in plants, where even short-term *in vitro* culture has been shown to act as an abiotic stress inducing TE activation [39-41].

Direct evidence for a possible role of TEs in genome structural variation was provided by the detection of multiple instances of TE-enriched CNV regions. In line with the view that TE silencing in FLM is less exhaustive than in FB, most amplified regions (96%) were found in free-living vegetative mycelia, where a significant fraction of the associated TEs was found to be transcriptionally active and hypomethylated. This suggests that a mechanism relying on hypomethylated TE copies, such as nonallelic (ectopic) recombination, may be responsible for the observed gain of genomic material.

On a more global scale, the results of genome-wide analyses of sequence similarity versus alignment length in different fungi also point to a less robust repeated element control in *T. melanosporum* compared to other fungi (Figure S13 in Additional file 1C). In fact, a broad and highly populated distribution centered around 200 to 900 bp, but extending to sizes larger than 4,000 bp, is observed in *Tuber*, whereas a much narrower and considerably less populated distribution, centered around 200 bp and rarely exceeding 2,000 bp, is present in the genomes of the RIP-proficient ascomycete *N. crassa*. In nearly all cases, both members of *Tuber* sequence pairs are methylated (>70%), with a trend toward higher methylation levels (>90%) for repeats smaller than 1,000 bp. Although genome-wide similarity analyses are not yet possible for *Ascobolus*, in which TEs comprise an estimated 14% of the genome, evidence derived from targeted experiments suggests very tight repeated element control also in this close relative of *Tuber* [21,42,43].

The extremely large fraction of the *Tuber* genome occupied by TEs - 6- to 20-fold higher than that of most filamentous fungi sequenced to date and about 4-fold higher than the relative TE content of *Arabidopsis* [44] - can itself be considered the result of a less comprehensive repeated element control. This suggests that incomplete TE methylation in *Tuber*, which may also be due to a less than perfect ‘maintenance methylation’, might reflect the inability to fully control such a massive amount of TEs, as indicated by the decrease of methylation levels observed also at symmetric CHG sites following 5-aza treatment. An alternative possibility is that somewhat leaky TE silencing may represent an evolutionary strategy aimed to support TE-mediated genome shaping and adaptation capacity. According to recent views on the generative role played by TEs in genome evolution [45], the latter situation might reflect the trade-off between the burden of TE maintenance and the possible role of such elements in promoting genome plasticity and the ability to adapt to sudden environmental changes [46,47]. It has also been proposed that genome shaping (for example, via CNV) and a robust adaptation capacity may be especially critical for organisms, such as plants, that cannot rely on behavioral responses to cope with stressful environments [45]. The same situation applies to fungi, especially plant pathogenic fungi and oomycetes that need to continuously evolve/update their host-pathogen interaction capacity [46], but may also be dramatically relevant in *Tuber* because of its hypogeous-plant symbiotic lifestyle and lack of an active spore dispersal system.

Truffles are not ideal model experimental organisms. However, they display a geographic area-restricted species distribution and because of their reputation as gastronomic delicacies, some of them, such as the highly prized black truffle *T. melanosporum*, are actively sought out and increasingly cultivated. This provides easy access to FB specimens from diverse habitats, which often exhibit distinct phenotypic features, including different volatile (‘aroma’) profiles [48]. It will thus be interesting to extend epigenomic surveys, such as the one presented in this work, to different *T. melanosporum* ecotypes. It will also be important to investigate the existence and possible functional significance of other TE silencing mechanisms (for example, RNA-induced post-transcriptional gene silencing) besides DNA methylation. Future studies in this direction may also help to explain the puzzling observation that TEs, but not protein-coding genes, have undergone a dramatic expansion in the *Tuber* genome.

## Conclusions

This work describes the selective methylation and associated transcriptional silencing of transposons in a complex fungal genome with massive repeat element content. Methylation profiles are highly similar amongst the three developmental stages that were investigated (FLM, FB and mycorrhiza), suggesting common, rather than tissue/stage-specific, roles for DNA methylation in truffles. Distinctive features of the *T. melanosporum* methylome compared to the few other fungi whose genome-wide methylation profiles have been determined so far are an extremely high level of cytosine methylation (>44% of total cytosine residues) with a marked preference for CG sites and large-sized (>1.5 kb) TEs. Along with other evidence, including the reduction of DNA methylation levels brought about by 5-aza treatment, this suggests that a reversible MIP-like process operates in truffles. Also remarkable is the non-exhaustive nature of TE methylation in *T. melanosporum*, which appears to be finely tuned with respect to genome location, in addition to TE size, with a clear trend toward hypomethylation for TEs located within a 1 kb distance from genes (especially highly expressed genes), rather than segregated in TE-rich regions of the genome. Indeed, over 300 hypomethylated or unmethylated TEs were found to be transcriptionally active, with higher expression levels in FLM compared to FB. Importantly, in FLM we also identified multiple instances of genome structural variations in the form of TE-enriched CNV regions bearing a significant fraction of hypomethylated and expressed TEs. This is likely due to prolonged *in vitro* culture, a stress known to stimulate TE activation in other organisms, and may represent novel evidence in favor of the postulated role of active TEs as genome plasticity and adaptation promoting elements. Non-exhaustive TE methylation and controlled TE activity, two features shared by various higher eukaryotic genomes, including the human genome, may be particularly important for the genome adaptation capacity of *Tuber* because of its hypogeous-plant symbiotic lifestyle and lack of an active spore dispersal system.

## Materials and methods

### Biological material

*T. melanosporum* (Vittad.) mycelium from the Mel28 strain, the same strain utilized for reference genome sequencing [24], was grown on 1% malt agar (Cristomalt-D, Difal, Villefranche-sur-Saône, France) for 5 weeks before harvesting. A mature FB collected in Auvergne was used for the FB library. ECM tips were from common hazel (*C. avellana* L.) plantlets inoculated with a *T. melanosporum* mycelium slurry (Raggi Vivai, Cesena, Italy*).* For 5-aza treatment, *T. melanosporum* mycelia (Mel28 strain) were grown in the dark at 23°C in synthetic liquid medium as described [49]. 5-Aza was added to *T. melanosporum* mycelia every 5 days at 10, 40, and 100 μM concentrations (from a 10 mM stock solution in water) for 45 days (‘treated’), with the last addition made 24 hours before harvesting of mycelia and DNA/RNA extraction. The same volume of water, instead of 5-aza, was added in parallel to control samples (‘untreated’).

### Library preparation and whole-genome bisulfite sequencing

Genomic DNA (gDNA) from FLM, FB and ECM was extracted by grinding in liquid nitrogen followed by DNeasy Plant Mini kit (QIAGEN, Hilden, Germany) purification. For nucleic acid extraction from 5-aza-treated and parallel control mycelia, tissues were ground in liquid nitrogen, extracted in 50% phenol-50% extraction buffer (100 mM Tris-HCl pH 8.0; 100 mM NaCl; 20 mM EDTA; 1% SDS) at 65°C for 10 minutes and centrifuged at 14,000 rpm for 10 minutes. The aqueous phase was transferred to new tubes and extracted twice with phenol:chloroform (1:1) and once with chloroform. Following ethanol precipitation, samples were resuspended in H_2_O. LiCl precipitation (2 M LiCl) was used to separate RNA (pellet) from gDNA (supernatant). gDNA was precipitated with ethanol and resuspended in H_2_O. Extracted gDNA was further purified with an additional phenol:chloroform extraction and sheared by sonication to generate DNA fragments in the 150 to 300 bp size range. Bisulfite treatment and library preparation were carried out as described [50], except that the EpiTect kit (QIAGEN) was utilized for bisulfite treatment. Two consecutive rounds of conversion were performed for a total of 10 hours. The resulting libraries were sequenced by Illumina sequencing technology (Illumina Hiseq 2000 sequencer; Illumina, San Diego, CA, USA). Reads were aligned to the reference genome (Tuber_melanosporum_v1.0) using BS Seeker [27]. Genome-wide DNA methylation profiles were generated by determining methylation levels for each cytosine in the genome. Since bisulfite treatment converts unmethylated cytosines (Cs) to thymines (Ts) after PCR amplication, the methylation level at each cytosine was estimated as #C/(#C + #T), where #C is the number of methylated reads and #T is the number of unmethylated reads. The methylation level per cytosine serves as an estimate of the percentage of cells bearing a methylated cytosine at a specific locus. We only included cytosines that are covered by at least four reads (except for the ECM methylome, which has a considerably lower overall coverage). The resulting methylation profiles per sample covered more than 90% of the cytosines.

### RNA-seq library preparation and data analysis

Total RNA was extracted as described above, dissolved in H_2_O after LiCl precipitation and purified with the RNeasy Plant Mini kit followed by on-column DNase I digestion (QIAGEN) as per the manufacturer’s instructions. RNA integrity was verified with a Bioanalyzer, which yielded RNA integrity number (RIN) scores of 7.0 and 6.5 for FB and FLM, respectively. RNA was quantified with the Qubit RNA BR Assay Kit (Life technologies, Carlsbad, CA, USA) and 1 μg of purified RNA was utilized as starting material for library construction, which was carried out with the Illumina TruSeq RNA Sample Preparation kit as per the manufacturer's instructions. Libraries were sequenced with a HiSeq 2000 System (Illumina) using 50-bp single-end reads. Reads were mapped against transcripts of genes and TEs using TopHat [51] allowing up to two mismatches, and only unique alignments were kept. The quality of alignments was checked using FastQC [52]. The resulting alignment files were processed with the HTSeq (version 0.5.4p3) [53] program to create a gene matrix for downstream analysis. Only reads mapping entirely to a single gene or TE were kept for further analysis. Reads corresponding to similar, but not identical, repeats were also mapped, albeit with a lower mapability compared to a regular gene alignment. In total, 63,742,213 and 74,109,065 reads from FB and FLM, respectively, were uniquely mapped, corresponding to an overall 93.34% and 84.88% mapping. Expression levels were computed as RPKM (Additional files 2 and 3). Gene length was calculated as the union length of all possible exons. An expression level of at least 1 RPKM was set as threshold for expressed genes and TEs [54].

### Phylogenetic analysis

Predicted amino acid sequences of putative DMTs from bacteria, viridiplanta and metazoa [7] and from a susbset of fungi were used for phylogenetic analysis. Accession numbers are reported in the Figure 7 legend, except for the sequences from *Aspergillus* spp. (*A. nidulans* (GenBank:EAA58167.1), *Aspergillus fumigatus* (GenBank:EDP47610.1), *Aspergillus niger* (GenBank:XP_001396716.1), *Aspergillus oryzae* (GenBank:BAE61916.1); viridiplanta (*A. thaliana* MET1 (GenBank:NP_199727) and CMT3 (GenBank:NP_177135.1), *Oryza sativa* MET1 (GenBank:B1Q3J6.1) and CMT3 (GenBank:NP_001049442.1), *Selaginella moellendorffii* MET1 (GenBank:EFJ35559.1), *Physcomitrella patens* MET1 (GenBank:XP_001758167), *Chlorella variabilis* MET1 (GenBank:EFN59320.1) and CMT3 (GenBank:EFN55234.1)); metazoa (*Mus musculus* DNMT1 (GenBank:P13864), puffer fish DNMT1 (Swiss-Prot:Q4RHE4), *Apis mellifera* Dnmt1 (GenBank:XP_001122269.1)); and bacteria (*Escherichia coli* EcoO109IM (GenBank:BAH79224.1), *Azoarcus* sp. BH72 (GenBank:YP_932275.1)). The third putative DMT of *A. immersus* (Masc3) was retrieved at the US Department of Energy Joint Genome Institute [55] by a BLASTP search using Masc1 and Masc2 as queries. Conserved domain composition was deduced by an RPS-BLAST search [56]. Phylogenetic analysis was performed with MEGA version 5 [57]. Amino acid sequences of predicted RID-like proteins and DMT catalytic domains were aligned with MUSCLE; maximum likelihood, using a substitution model with evolutionary rate variation among sites (Gamma distributed with five categories), was employed for phylogeny reconstruction. A parallel phylogenetic analysis, which yielded an essentially identical tree topology for both RID-like and DMT-like proteins, was conducted with neighbor-joining using uniform rates among sites. The bootstrap method of interior-branch test [58] was used to assess tree reliability.

### Genome annotation

The *T. melanosporum* reference genome sequence utilized for the present study (NCBI reference sequence: NZ_CABJ00000000.1) refers to assembly v_1.0. Annotations were downloaded from MycorWeb INRA-Nancy [59]. TEs were classified as in [24] with minor modifications. Repeat annotations for *N. crassa*, *U. reesii, A. nidulans*, and *S. cerevisiae* were predicted from the corresponding genome sequences [60-63] by RepeatMasker [64].

### Other procedures

Differentially methylated regions were identified by first searching for TEs with significantly different methylation levels between the FLM and FB methylomes. TEs with at least four CG or non-CG (CHG, CHH) sites in both samples were selected for differential methylation analysis. For each CG site, we calculated the delta methylation (∆m), that is, the difference in methylation levels between the two groups. We then calculated the z score of the average ∆m level for each TE as a measure of differential methylation within a given TE. A TE was considered as differentially methylated if the absolute z score value was larger than 1.96 and mean methylation levels between the two samples differed by at least 20% (Figure S2 in Additional file 1). Differentially methylated genes were identified in the same way, except for a reduced (10%) difference between the mean methylation levels of the two samples, to take into account the generally much weaker methylation of genes compared to TEs.

The CRI was calculated as (RIP product) - (RIP substrate) [33]. The RIP product score $\left(\frac{TpA}{ApT}\right)$ measures the frequency of RIP products taking into account potential false positives due to local density, while the RIP substrate score $\left(\frac{CpA+ TpG}{ApC+ GpT}\right)$ measures the depletion of RIP targets and their reverse complement (for example, CpA and TpG). Computing CRI details, including raw data, processed data, scripts (python and R) and output files are available in Additional files 4 and 5 and can be accessed at [65].

### Data access

Methylation and RNA-seq data can be accessed at the UCSC Genome Browser [66] and at Gene Expression Omnibus (GSE49700).

## Additional files

Additional file 1
**Supplementary Tables S1 to S4 and all supplementary figures.**
Additional file 2
**Table S5, which lists TE methylation and expression data.**
Additional file 3
**Table S6, which lists gene methylation and expression data.**
Additional file 4
**The source Python and R scripts as well as a brief manual for CRI computation.**
Additional file 5
**The 10 output CRI files used to generate the plots shown in Figure S13A,B in Additional file** 1 **.**

## Abbreviations

5-aza: 5-azacytidine
5mC: 5-methylcytosine
bp: base pair
BS-seq: bisulfite sequencing
CMT: chromomethyltransferase
CNV: copy number variation
CRI: composite RIP index
DMT: DNA methyltransferase
ECM: ectomycorrhiza
FB: fruitbody
FLM: free-living mycelium
gDNA: genomic DNA
LINE: Long interspersed nuclear element
LTR: long terminal repeat
MIP: methylation induced premeiotically
RIP: repeat-induced point mutation
RPKM: reads per kilobase per million mapped reads
SNP: single-nucleotide polymorphism
TE: transposable element
