# Supplementary material for: Non-exhaustive DNA methylation-mediated transposon silencing in the black truffle genome, a complex fungal genome with massive repeat element content
Source: Genome Biol. 2014 Jul 31;15(8):411. doi: 10.1186/s13059-014-0411-5 (PMC4165359; doi:10.1186/s13059-014-0411-5)
Supplement: Additional file 1 — Supplementary Tables S1 to S4 and all supplementary figures. [file 13059_2014_411_MOESM1_ESM.docx]

## Table S1. Sequence and mapping data for bisulfite-treated (BS-seq) and non-bisulfite-treated (WG-seq) genomic DNA.

| sample | library/ strategy | # raw reads | # uniquely mapped | mapability | read length | coverage per strand (X) |
| --- | --- | --- | --- | --- | --- | --- |
| FLM | BS-seq | 122,903,319 | 78,983,505 | 64.26% | 100 | 31.61 |
| FB | BS-seq | 184,692,678 | 86,778,157 | 46.99% | 100 | 34.73 |
| ECM | BS-seq | 182,286,685 | 3,268,721 | 1.79% | 90 | 1.18 |
| 5-aza treated | BS-seq | 58,665,936 | 41,349,427 | 70.48% | 51 | 8.44 |
| 5-aza untreated | BS-seq | 50,362,313 | 35,348,536 | 70.19% | 51 | 7.21 |
| FLM | WG-seq | 57,386,669 | 45,239,191 | 78.83% | 51 | 9.23 |
| FB | WG-seq | 70,844,918 | 50,646,551 | 71.49% | 51 | 10.34 |

### Table S2. Bulk methylation levels: “common cytosines” shared by FB and FLM, and FB, FLM and ECM.

|  | **CG** | **CHG** | **CHH** |
| --- | --- | --- | --- |
| 49M common sites shared by FB and FLM | | | |
| FB | 30.8% | 12.1% | 10.6% |
| FLM | 30.2% | 9.8% | 9.9% |
| 260K common sites shared by FB, FLM, and ECM | | | |
| FB | 8.7% | 5.2% | 3.7% |
| FLM | 7.8% | 3.4% | 2.8% |
| ECM | 8.4% | 4.7% | 3.5% |

### Table S3. Bulk methylation levels: total “common cytosines” shared by untreated and 5-aza-treated genomic DNA from free-living mycelia.

|  | **CG** | **CHG** | **CHH** |
| --- | --- | --- | --- |
| 36M common sites shared by  5-aza treated and untreated samples |  |  |  |
| 5-aza untreated | 23.62% | 7.98% | 9.46% |
| 5-aza treated | 23.57% | 6.59% | 7.87% |

### Table S4. Bulk methylation levels: TE “common cytosines” shared by untreated and 5-aza-treated FLM genomic DNA.

|  | **CG** | **CHG** | **CHH** |
| --- | --- | --- | --- |
| 11M common sites within TEs shared by  5-aza treated and untreated samples |  |  |  |
| 5-aza untreated | 77.76% | 17.33% | 20.06% |
| 5-aza treated | 77.62% | 14.28% | 16.69% |


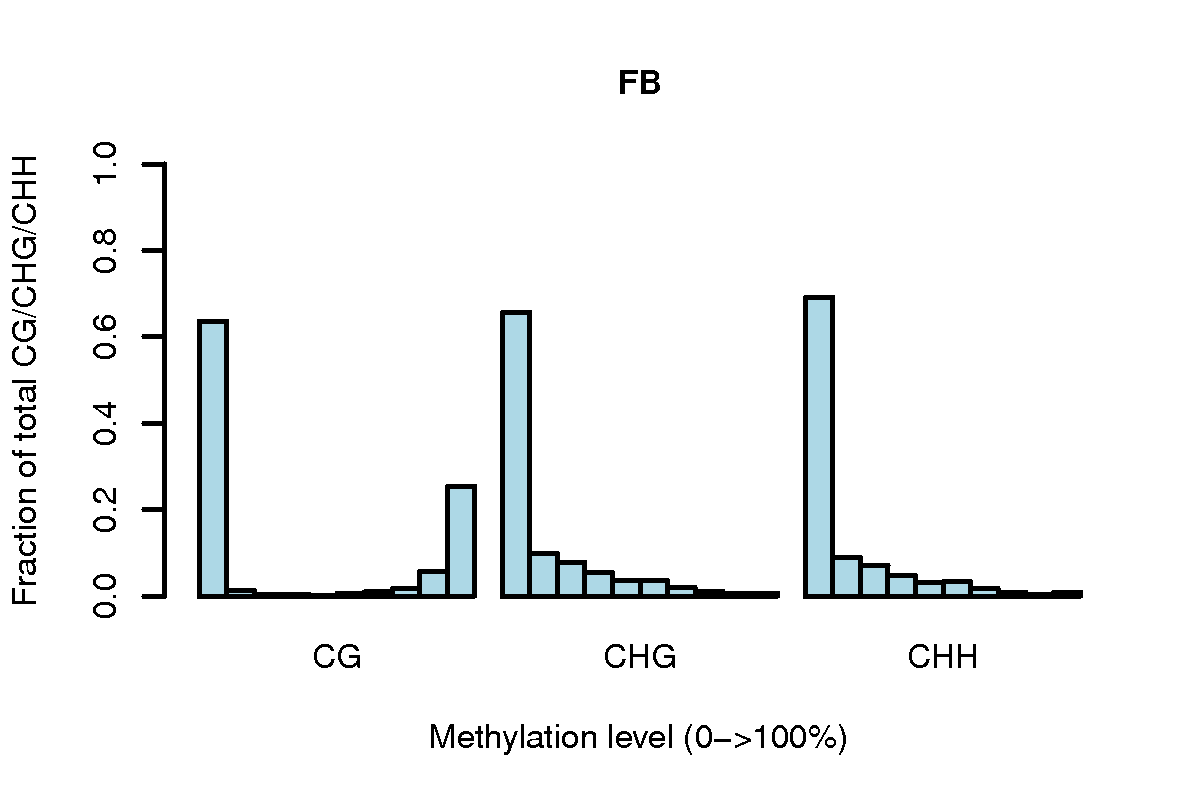

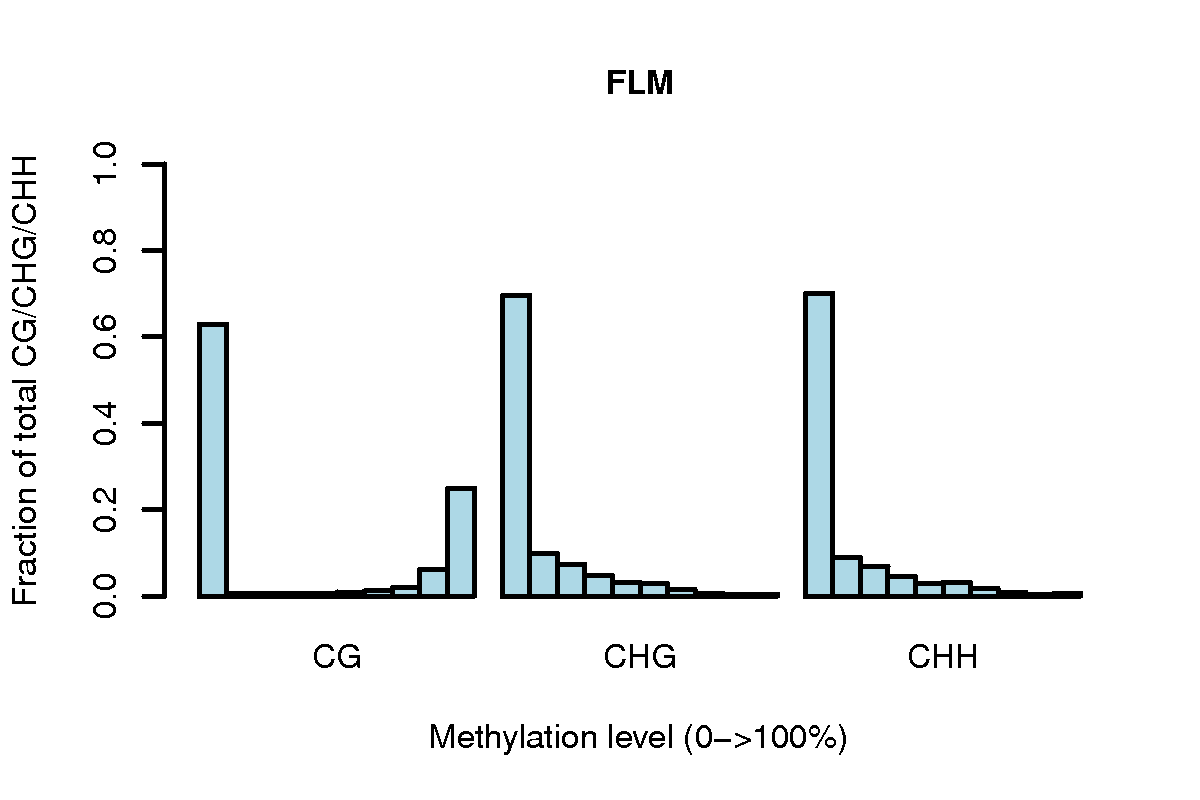

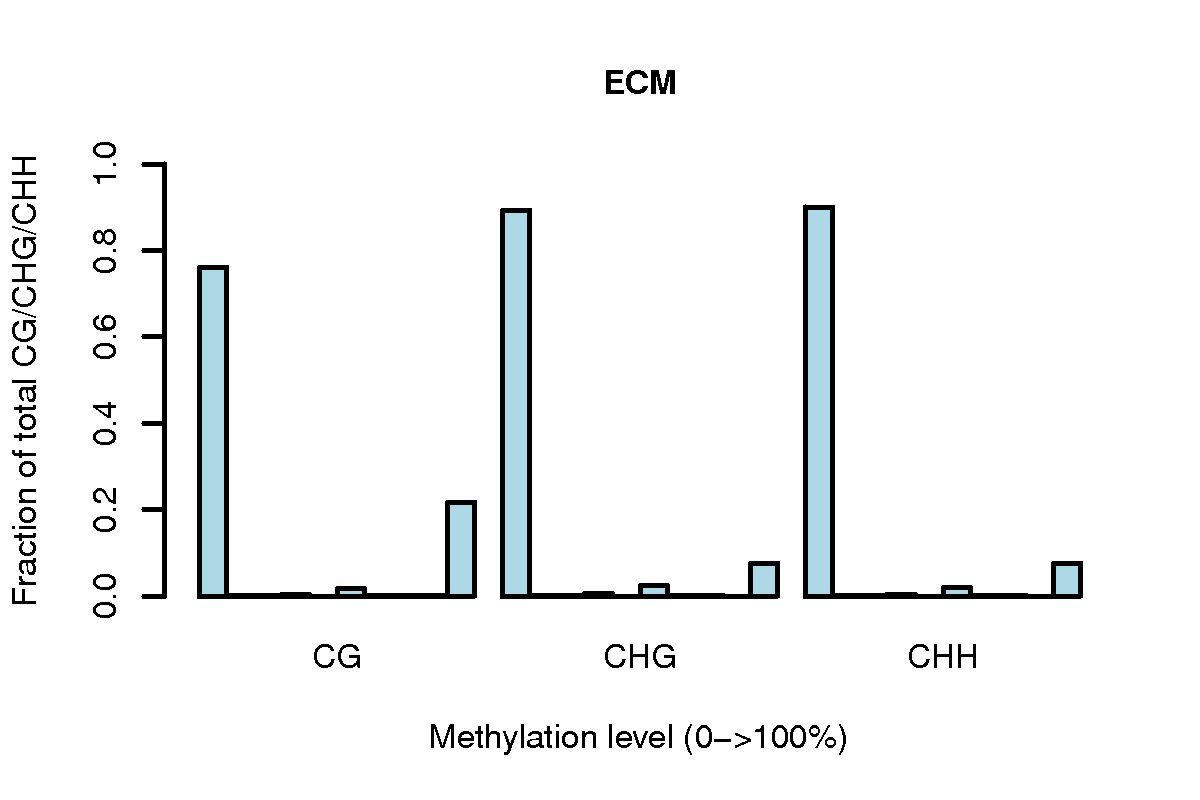


### Figure S1. Histogram representation of DNA methylation levels (0-100%) in FB, FLM, and ECM as a function of sequence context (CG, CHG, and CHH).


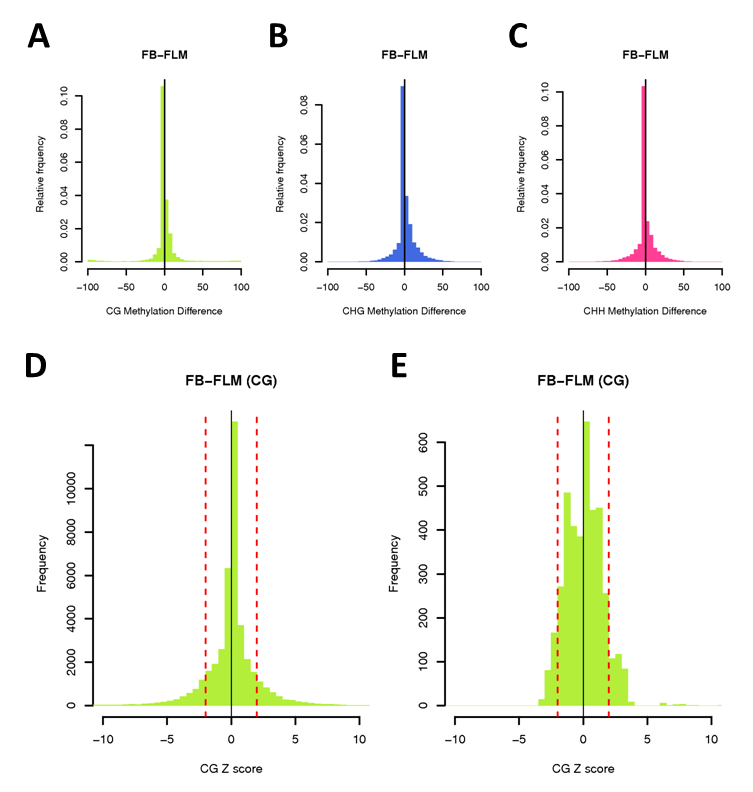


**Figure S2.** Histogram of ∆ methylation levels (FB-FLM). Average ∆ methylation of FB vs. FLM was 0.57 (**A**), 2.32 (**B**) and 0.77% (**C**) for CG, CHG, and CHH sites, respectively. The distributions of standardized Z scores for ∆ methylation levels (FB vs. FLM) within TEs and genes are shown in panels (**D**) and (**E**).


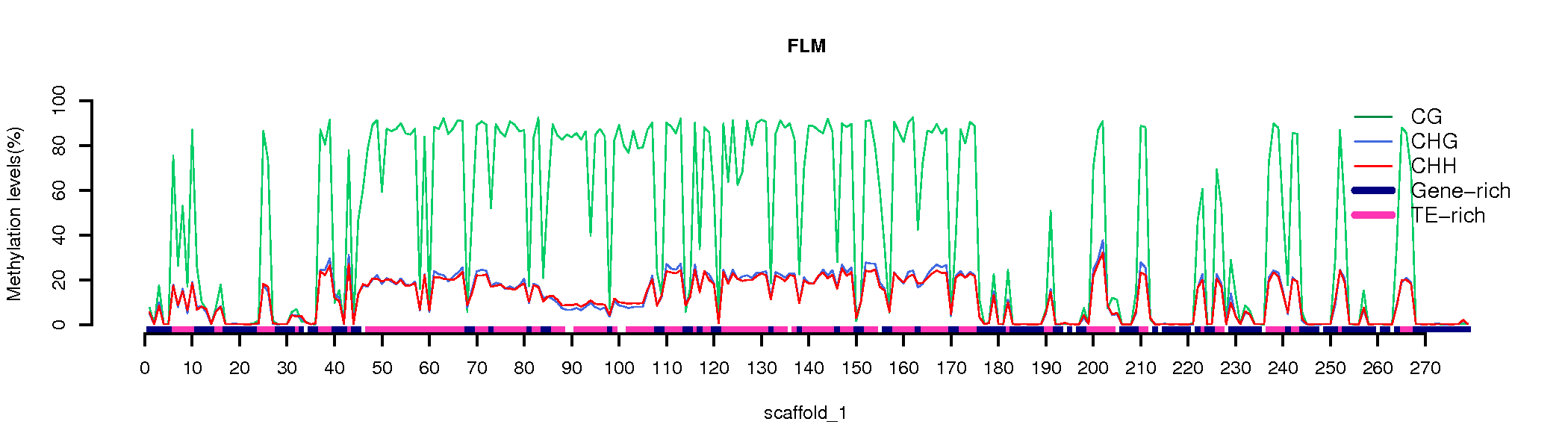

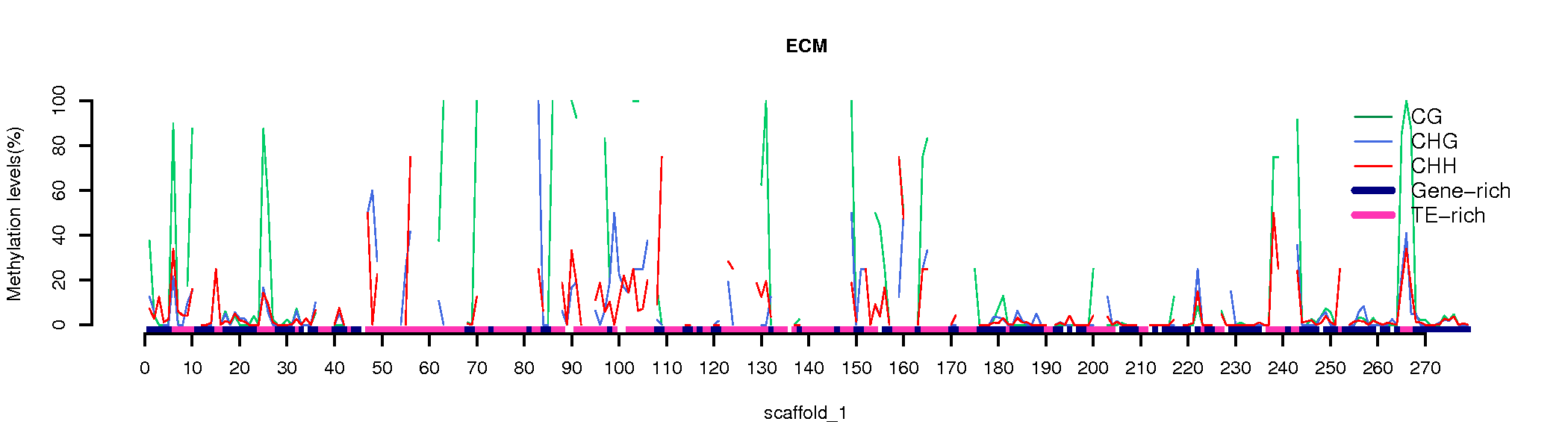


**Figure S3.** Large-scale view (scaffold 1) of DNA methylation levels in FLM and ECM. Methylation levels at CG, CHG, and CHH sites are plotted along scaffold 1 for FLM (*top*) and ECM (*bottom*); gene- or TE–rich regions are shown in the bottom tracks.


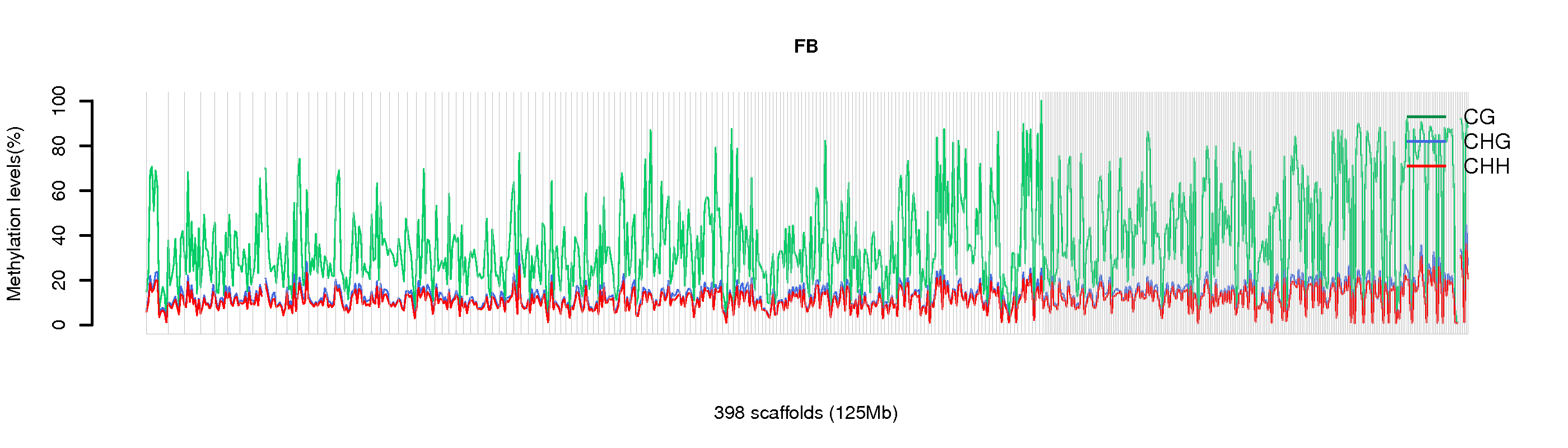

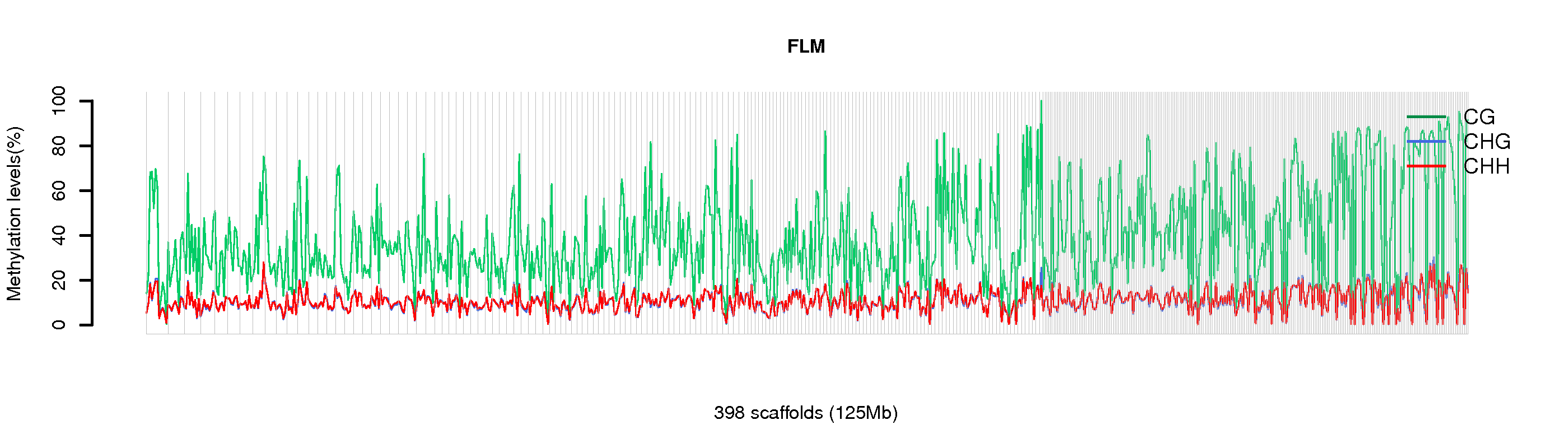

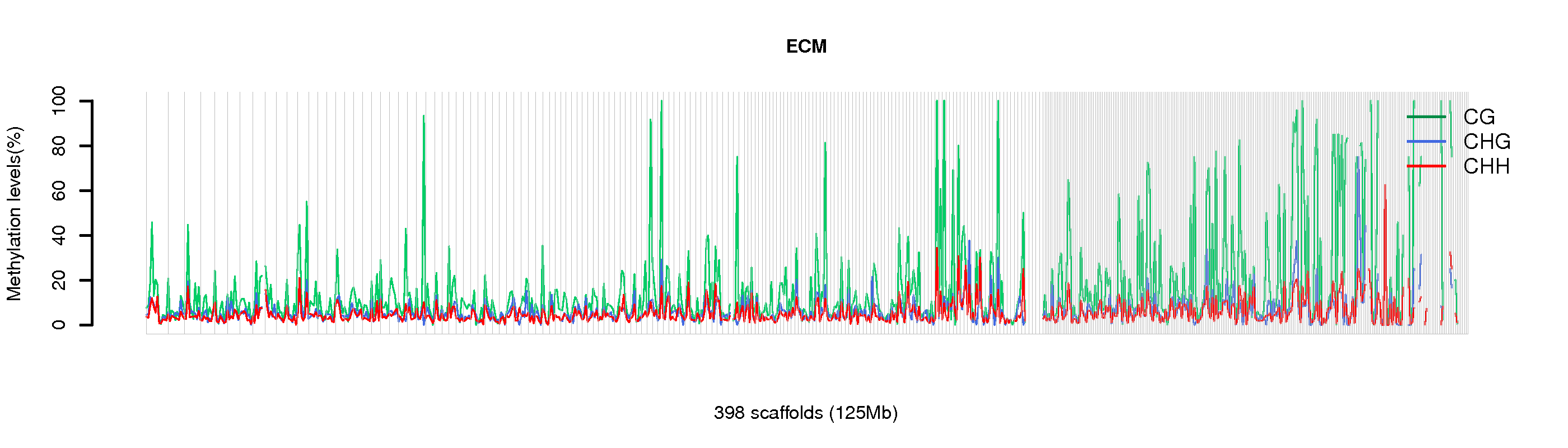


**Figure S4.** Genome-wide view of DNA methylation levels at CG, CHG and CHH sites in FB (*top*), FLM (*middle*) and ECM (*bottom*).


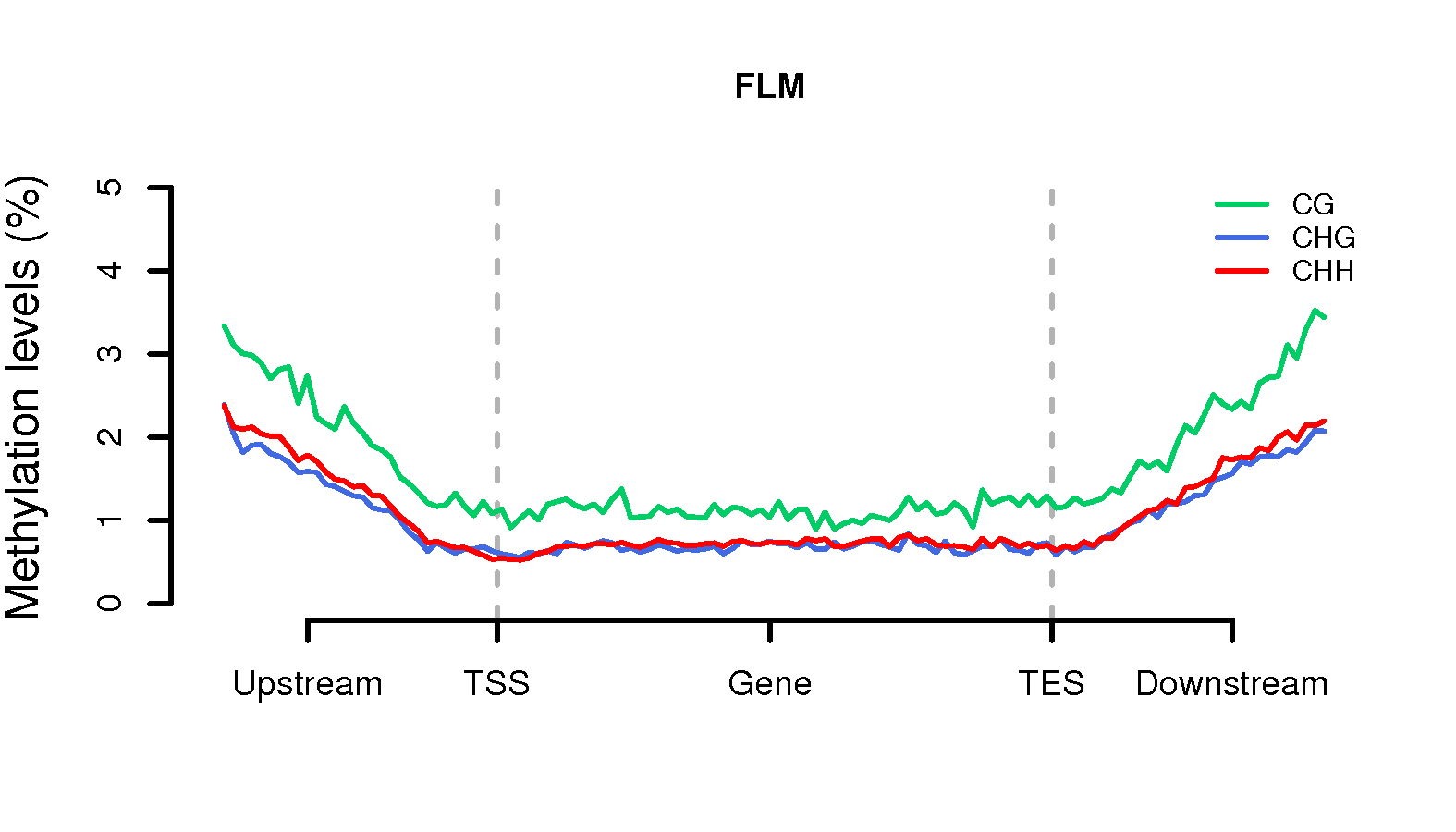

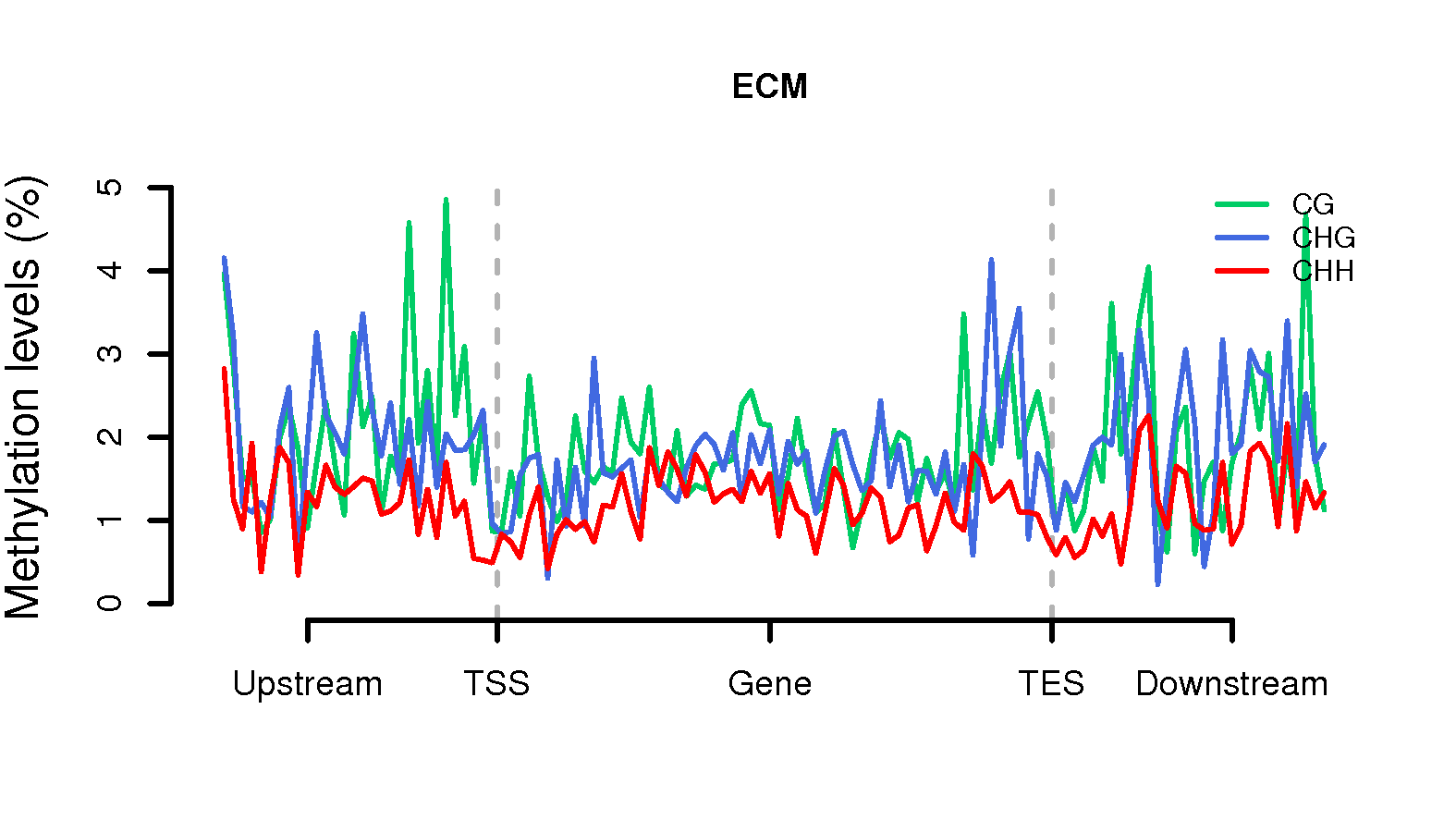

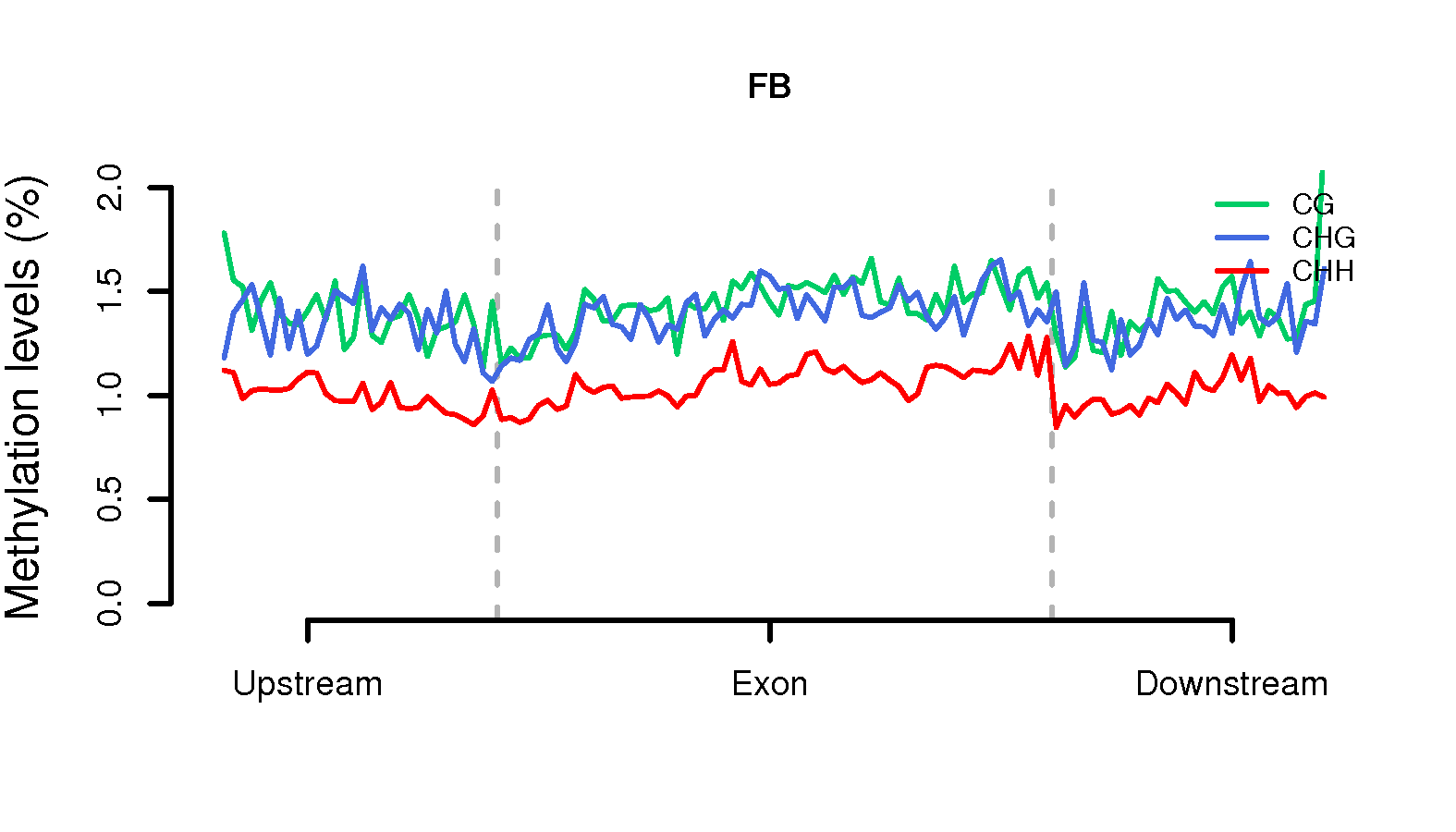

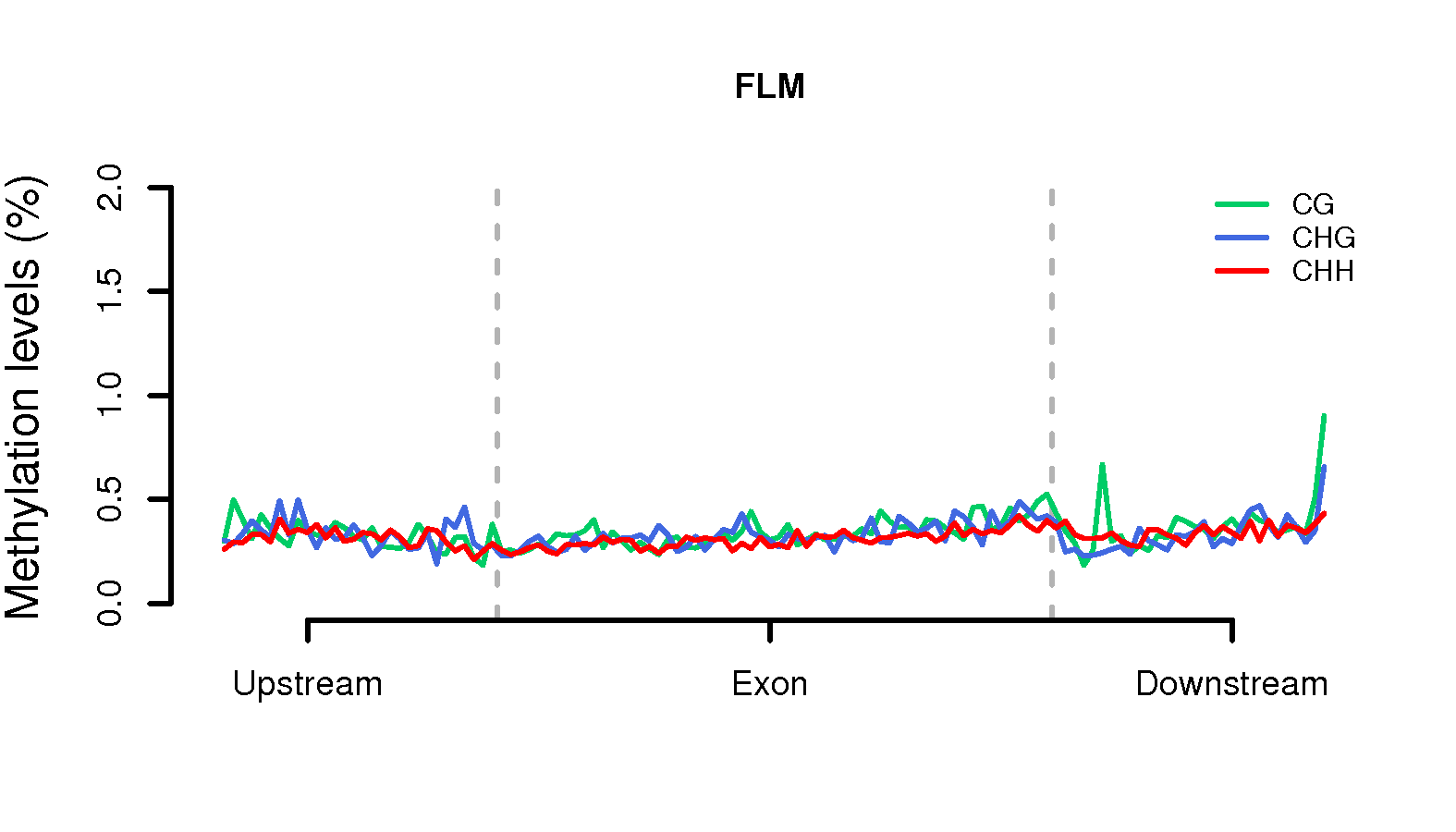


**Figure S5.** Meta-plots of DNA methylation levels in genes (*top panels*: FLM and ECM) and exons (*bottom panels*: FB and FLM). Plotted values are average methylation levels within upstream, core genomic (genes, exons) and downstream regions.


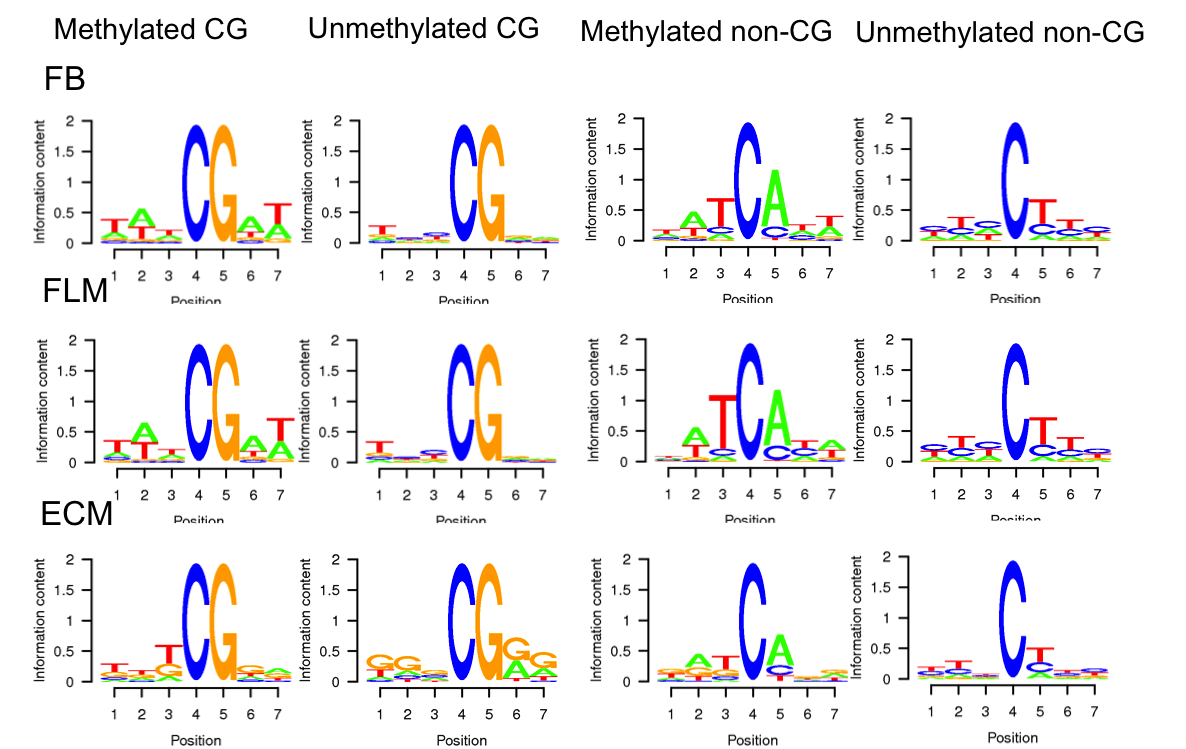


**Figure S6.** Logo-plots of sequences proximal to methylated and unmethylated sites in CG and non-CG sequence contexts.


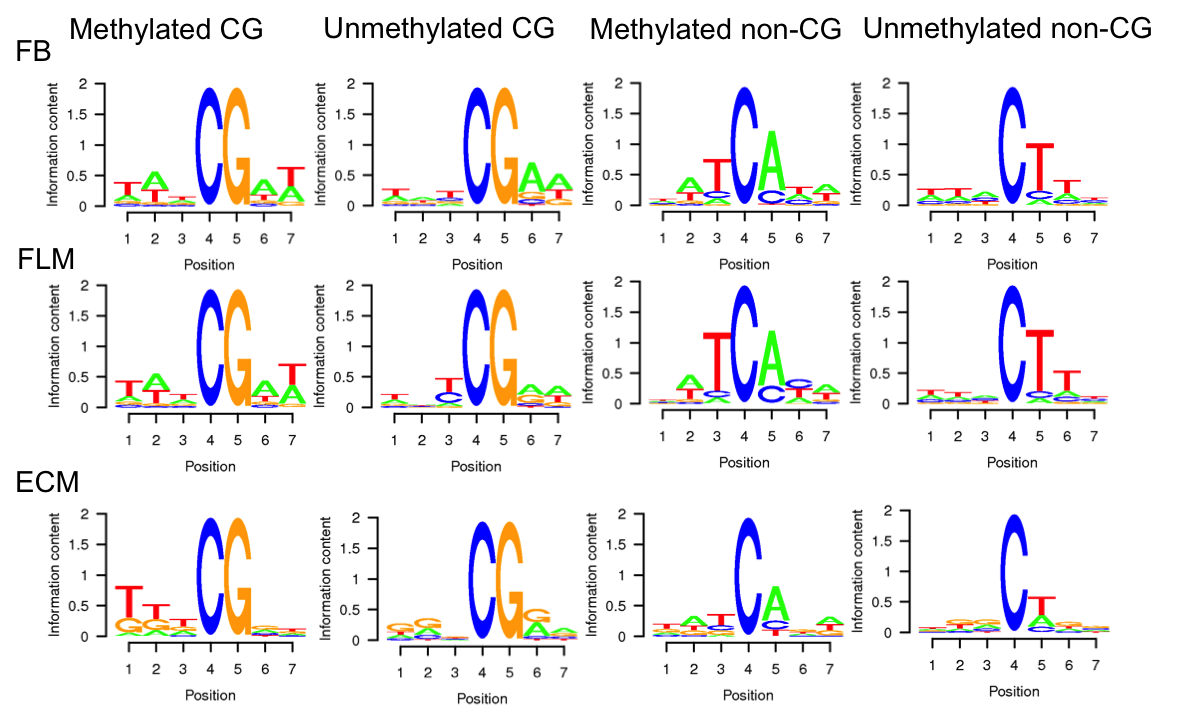


**Figure S7.** Logo-plots of sequences proximal to transposon-associated, methylated and unmethylated sites within CG and non-CG sequence contexts.

***
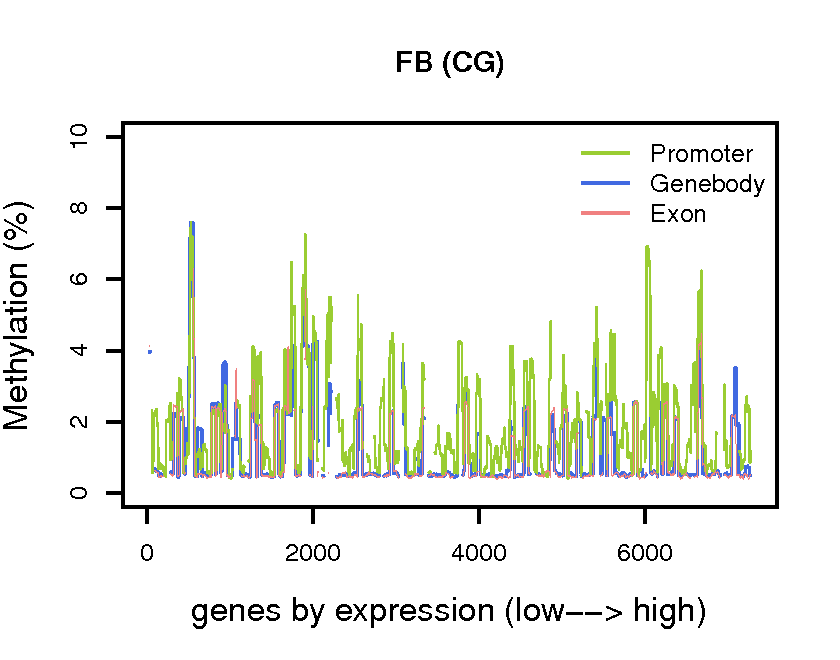

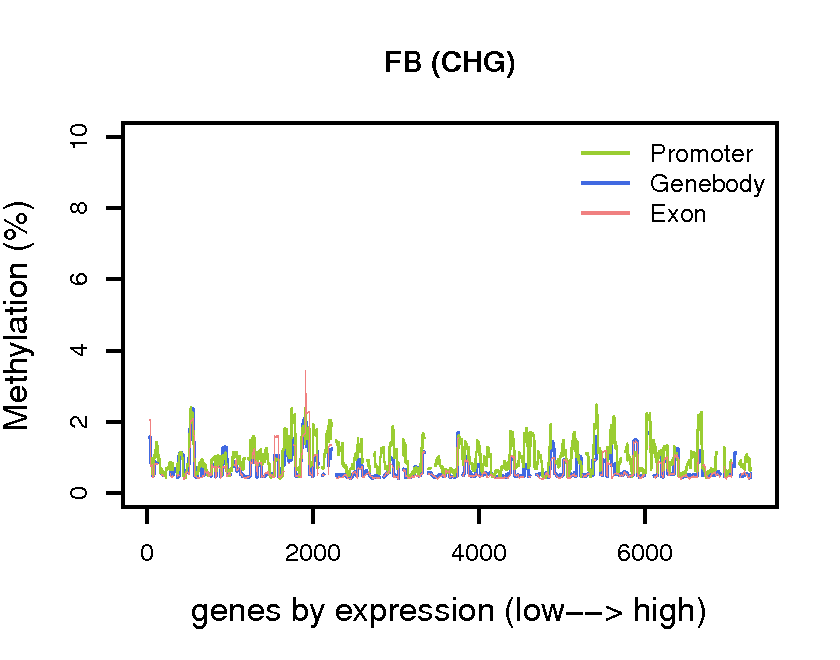

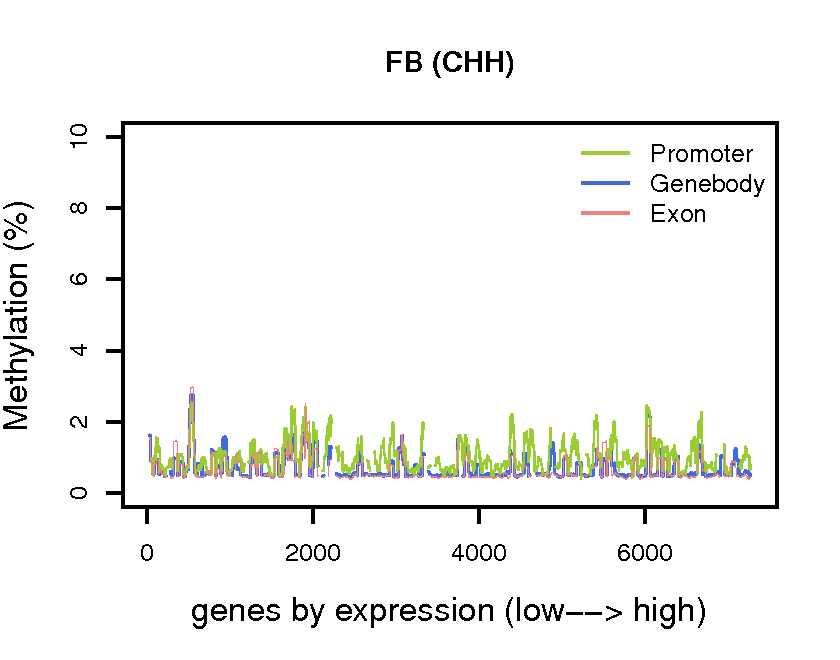
***

***
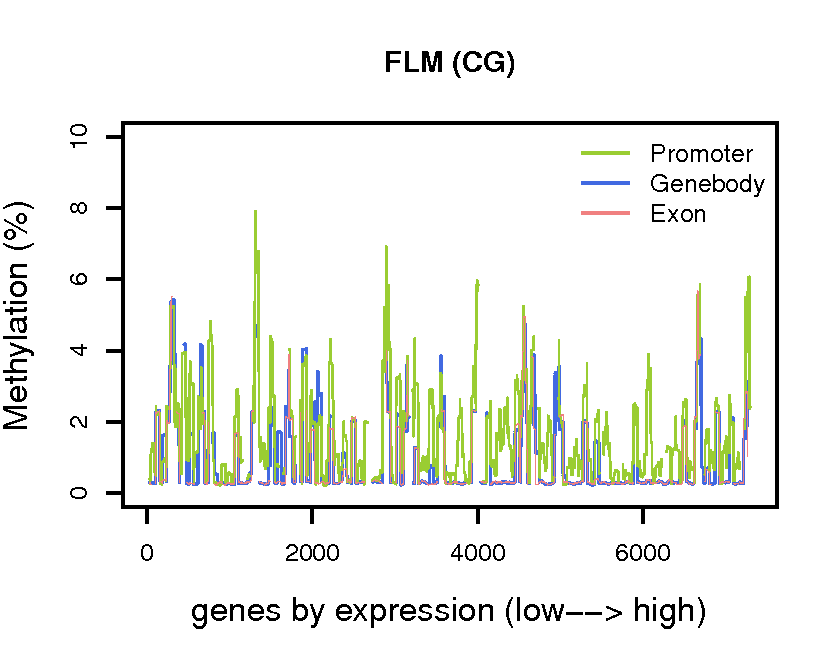

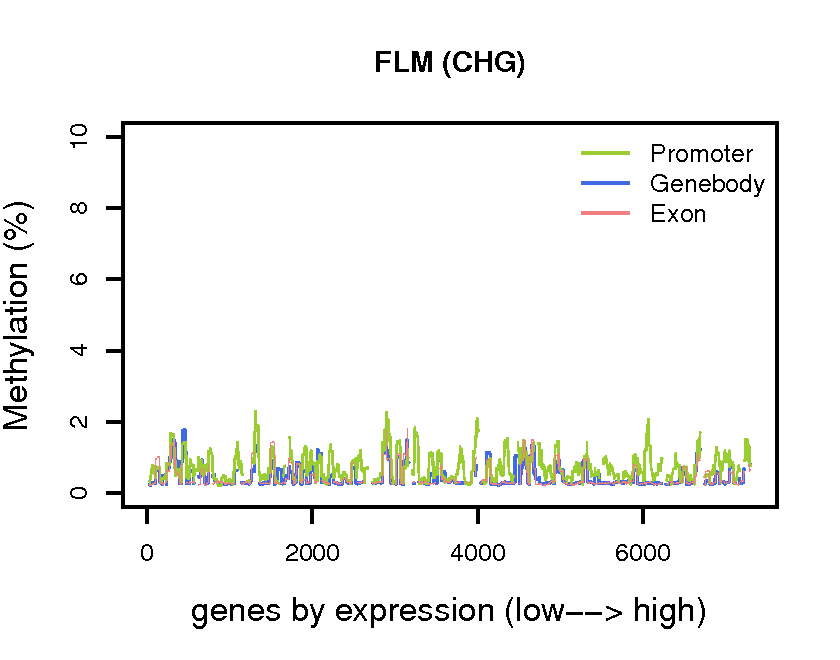

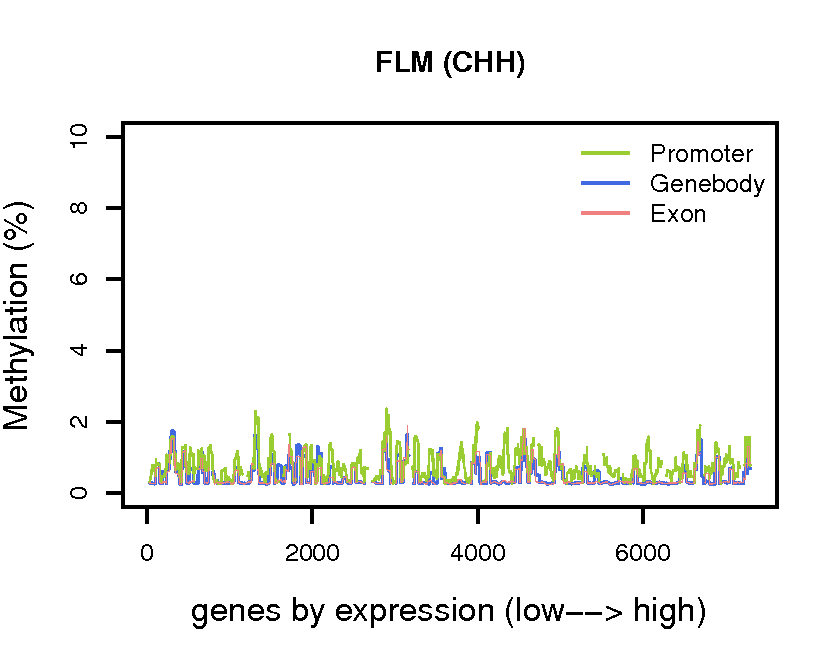
***

**Figure S8.** Promoter and gene-body methylation levels ranked according to gene expression levels (low to high); methylation levels are plotted as moving averages of 50 genes.

**
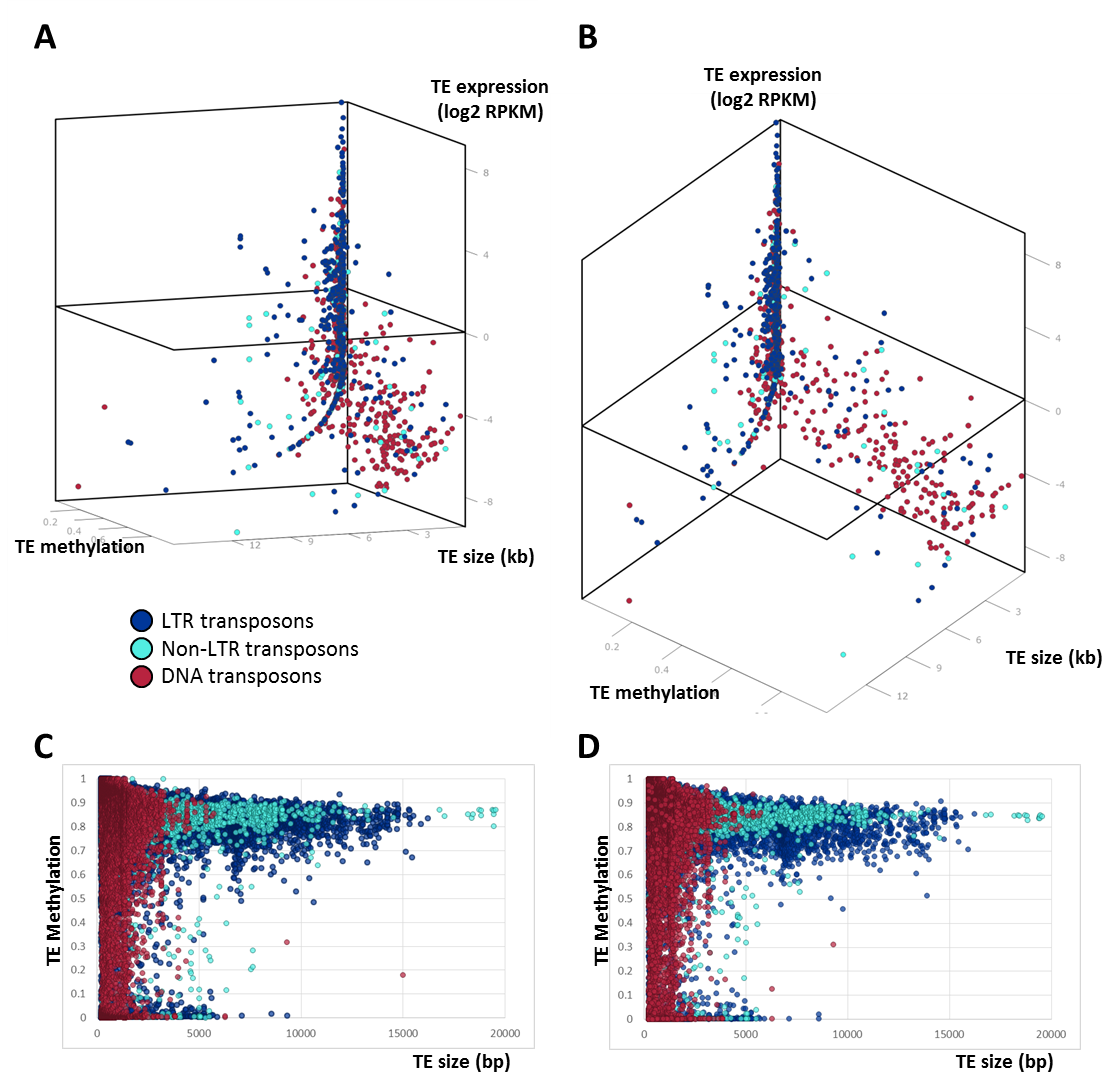
**

**Figure S9.** Transposon methylation and expression. CG methylation levels of individual TEs plotted against their size (kb) and expression levels (Log2 RPKM) in FLM (**A-B**) presented as two views of the same three-dimensional scatter plot. Only TEs with at least one mapped read (RPKM>0) are shown. The plane cut at log2 RPKM= 0 was set as a threshold for expressed TEs (corresponding to RPKM=1); see also Figure 2 (main text). CG methylation levels vs. size (bp) of TEs with RPKM=0 are presented as scatter plots for FB and FLM in panels (**D**) and (**E**)**.** Retrotransposons are shown in blue (dark blue for LTR, light blue for non-LTR retrotransposons); DNA transposons are shown in red.

**A.**


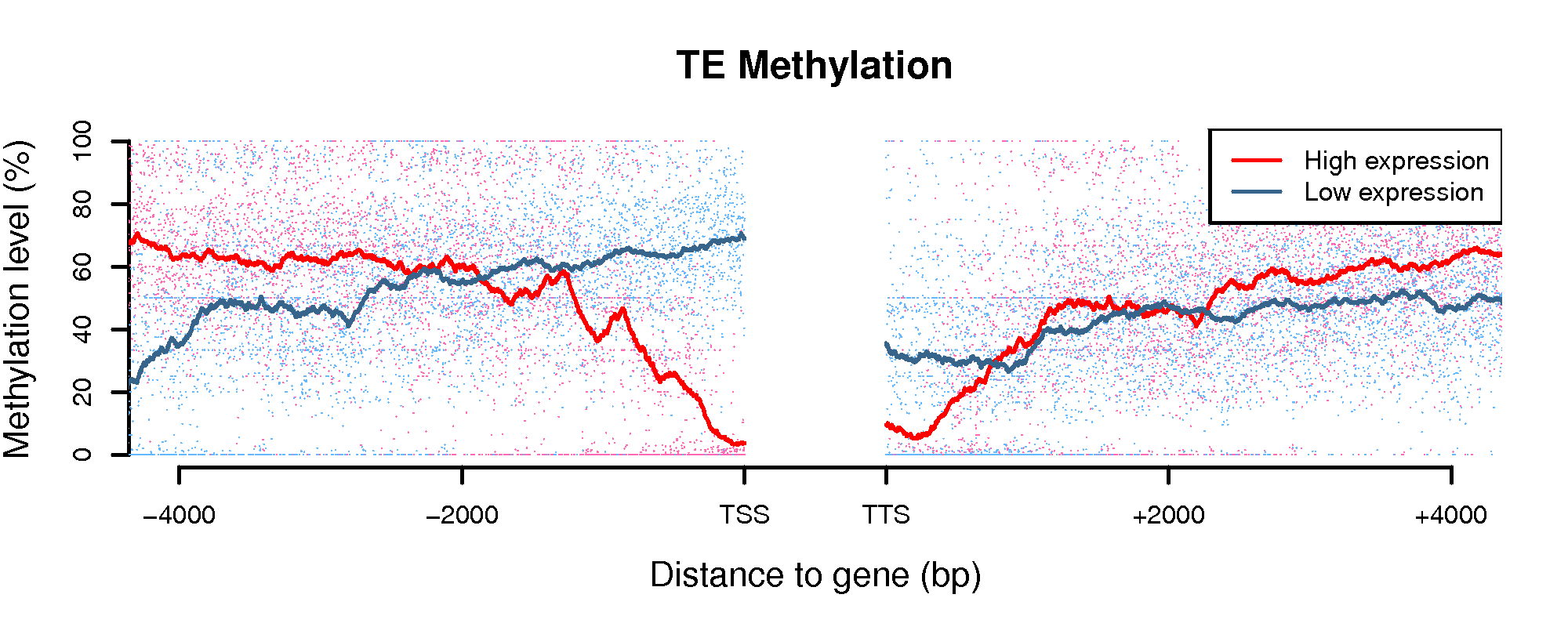


**B.**


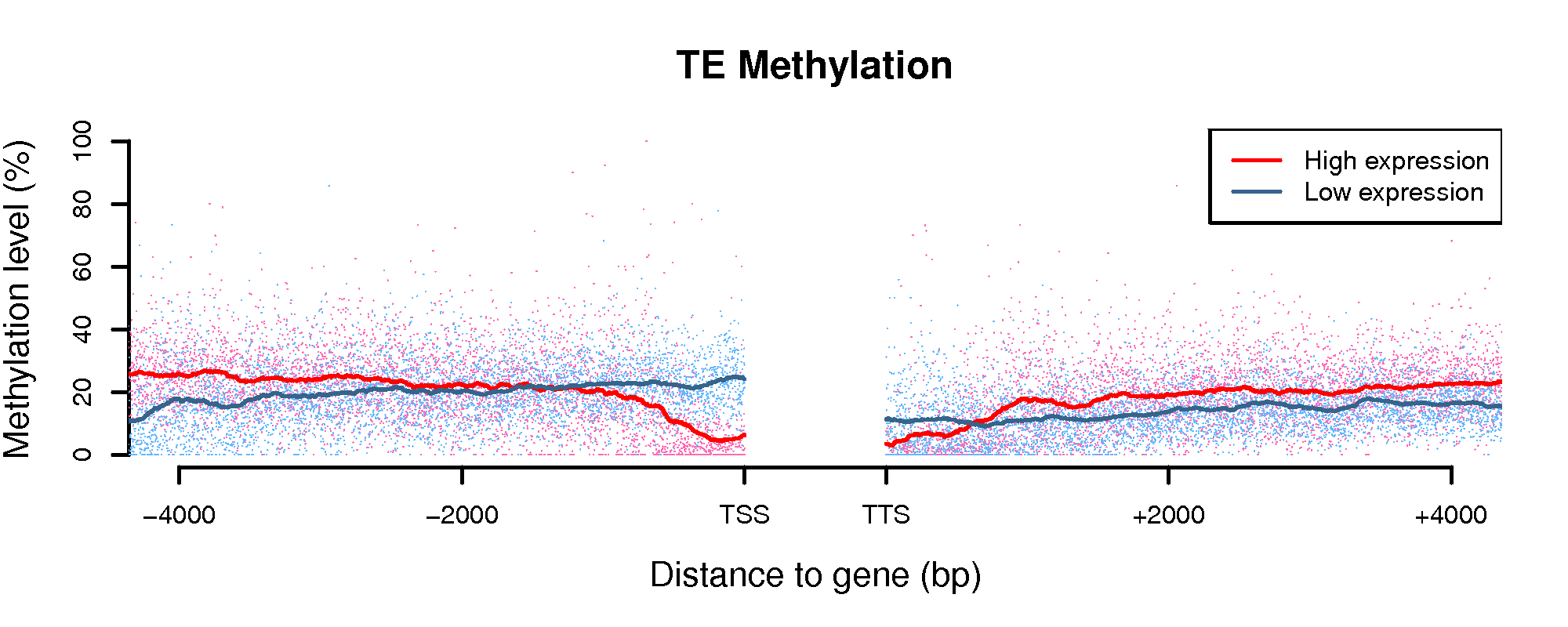


**C.**


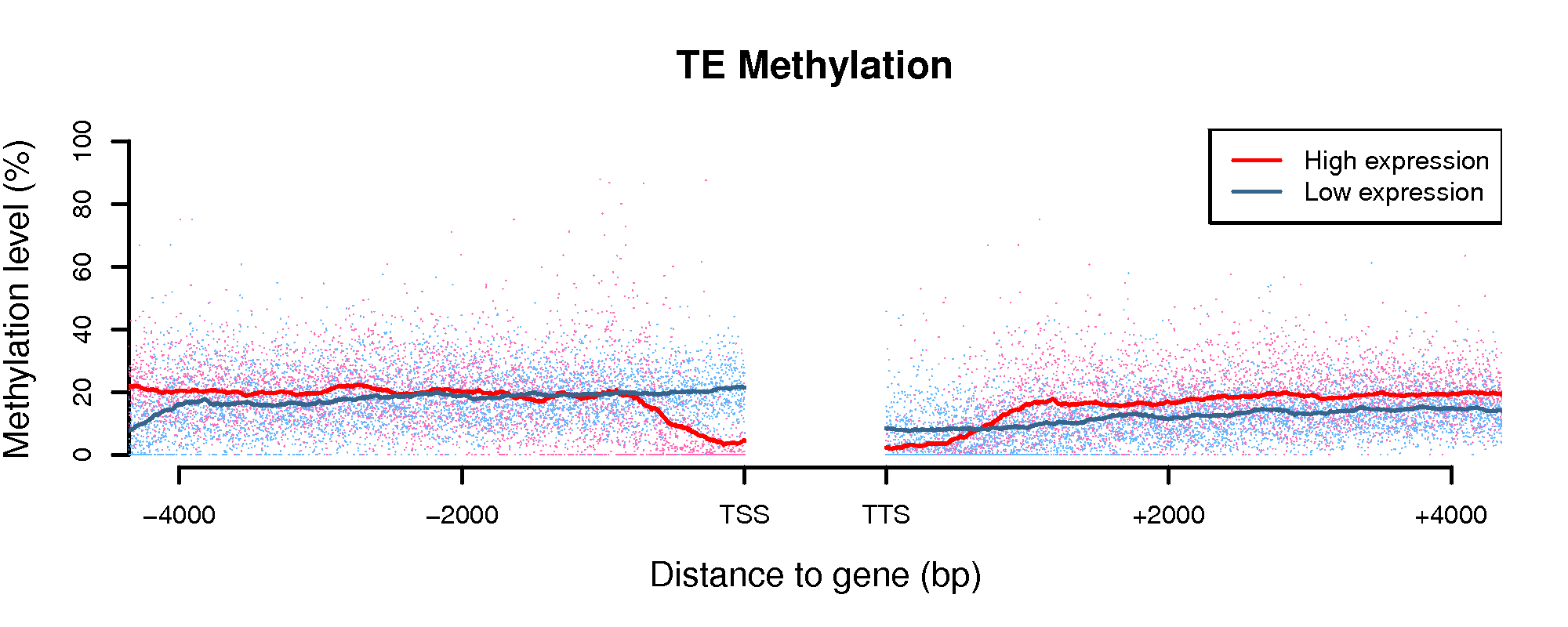


**Figure S10.** Transposon methylation and neighbor gene expression levels for FLM (CG sites), FB (CHG sites) and FLM (CHG sites) are shown in panels (**A**), (**B**) and (**C**), respectively. Methylation levels are referred to TEs located upstream or downstream to highly (top 25%) or lowly (bottom 25%) expressed genes. *Red* (highly expressed genes) and *blue* (lowly expressed genes) lines represent the moving average of methylation levels in 100 bp windows. t-test *p*-values of the differences in % TE methylation between highly and lowly expressed genes were 2.2∙10^-16^ for TE-gene distances ≤0.5kb and ≤10^-4^ for TE-gene distances comprised between 0.5 and 1 kb.

**A.**

**
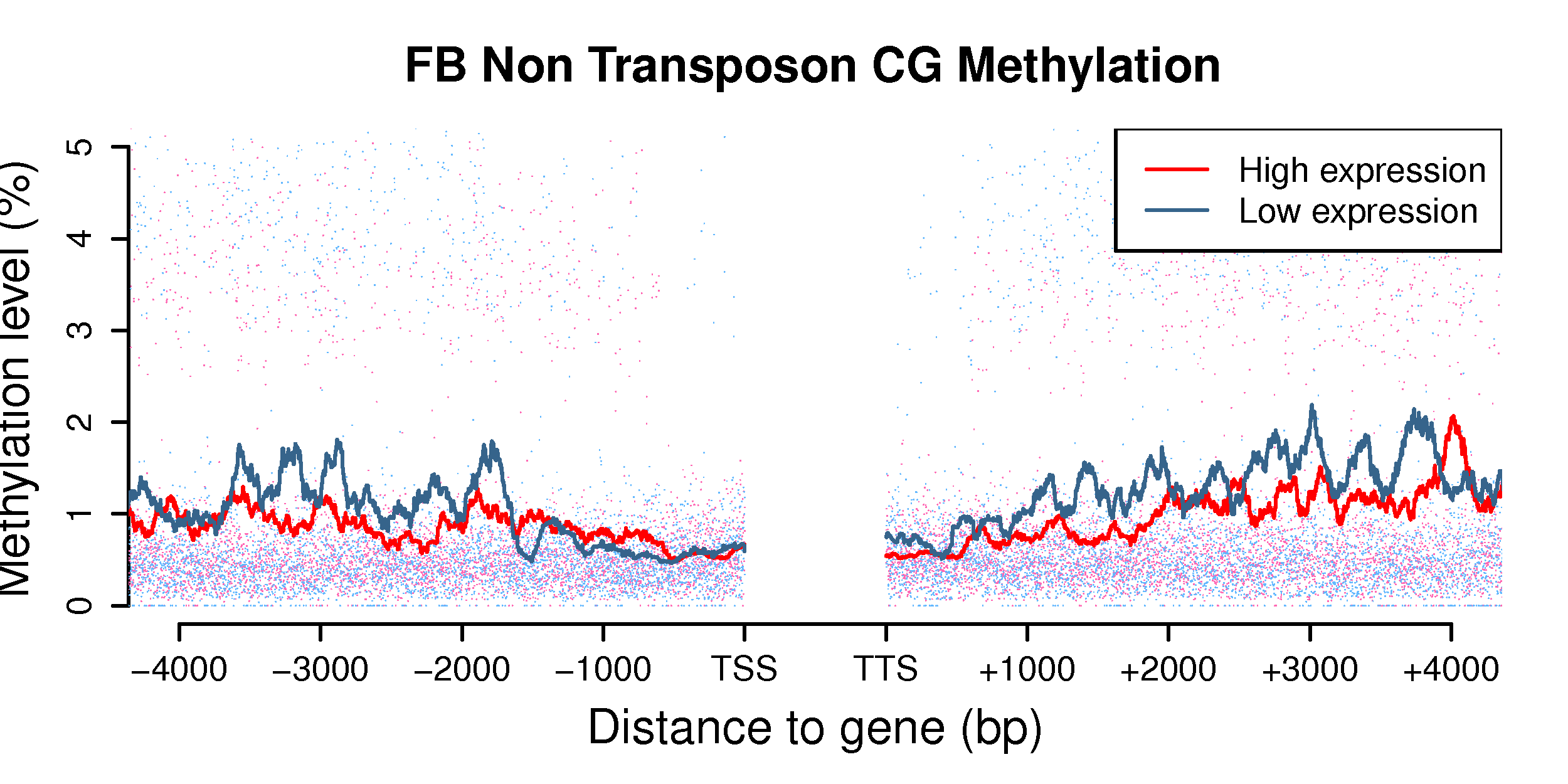
**

**B.**

**
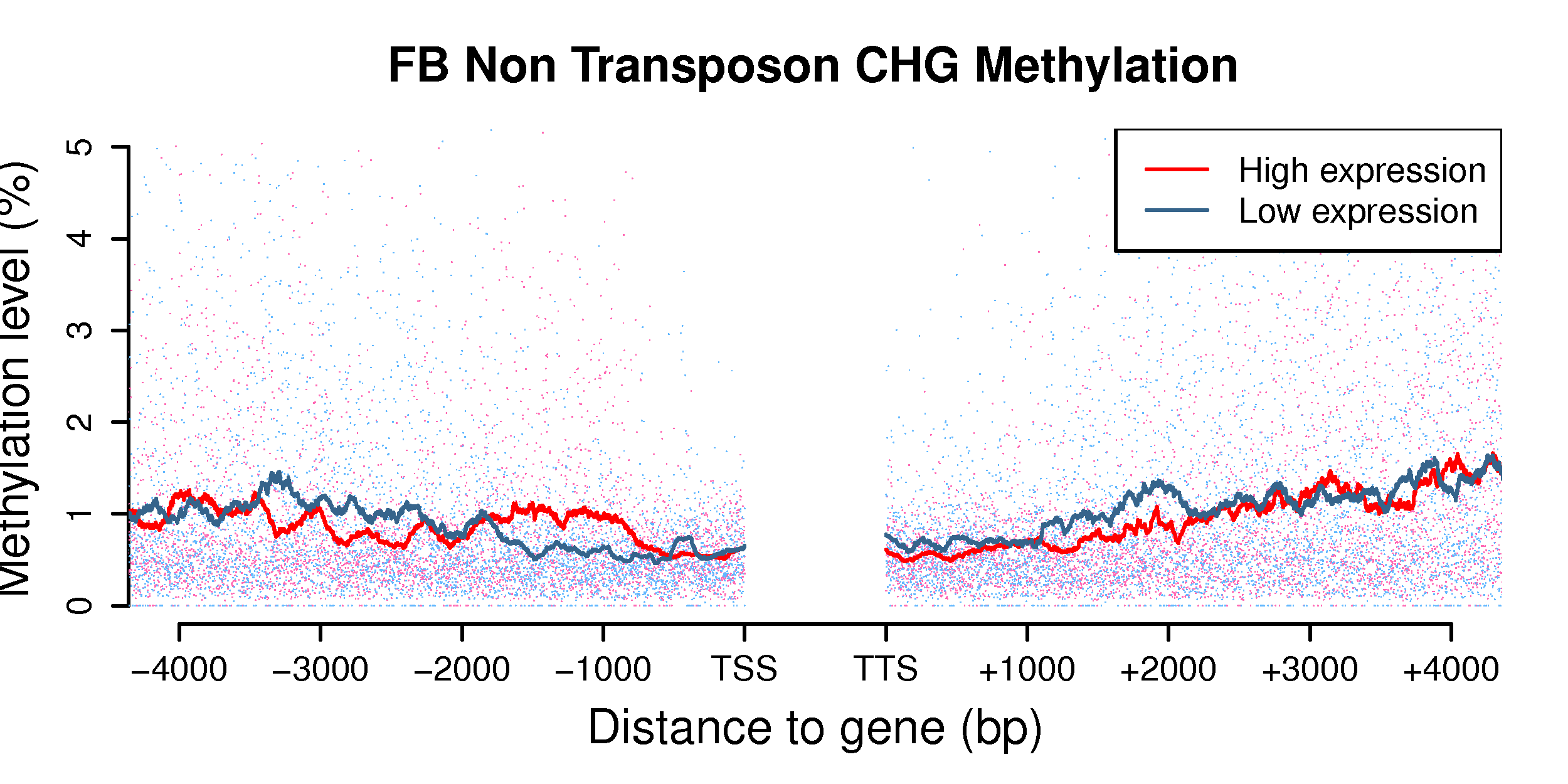
**

**C.**

**
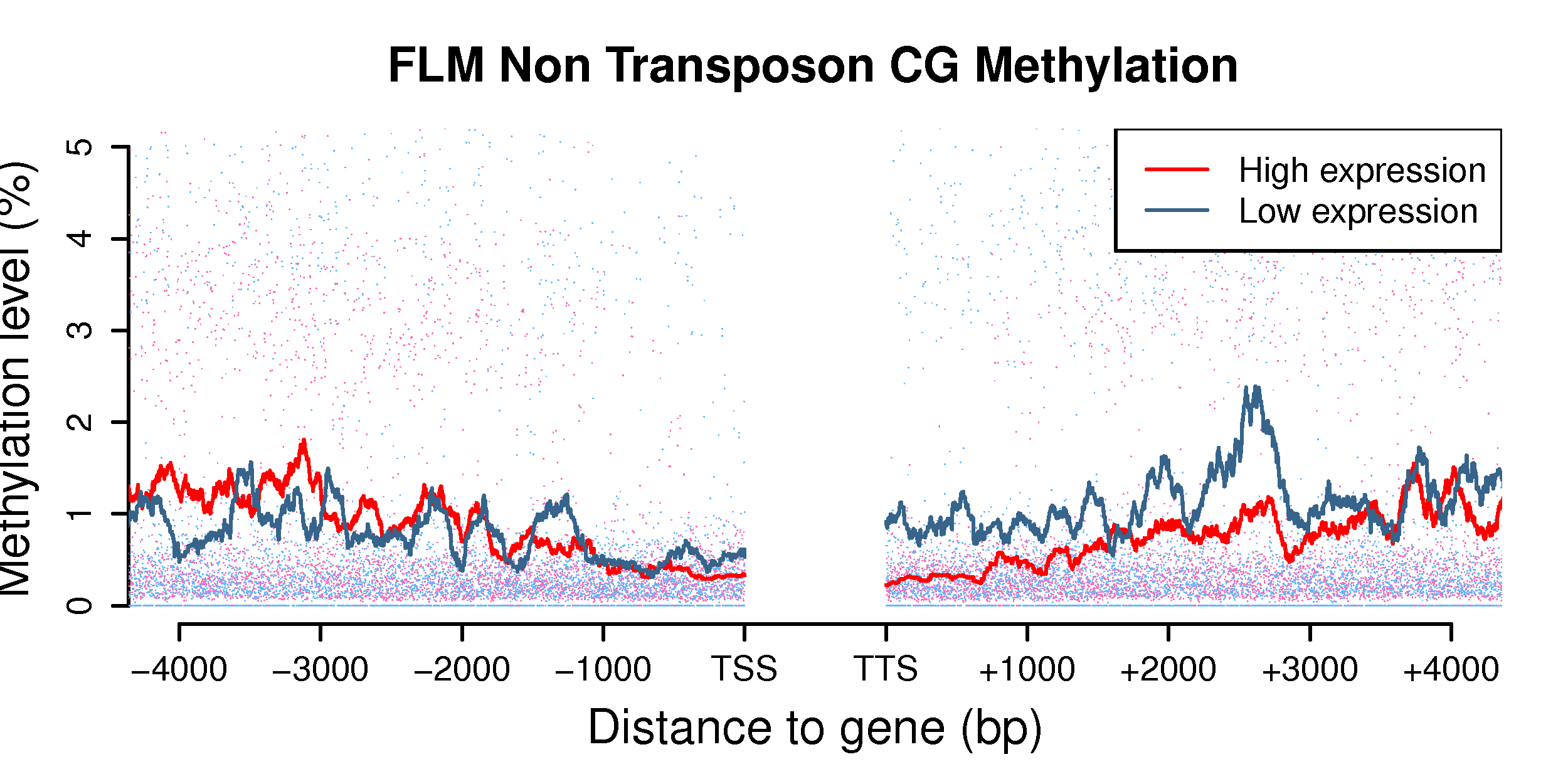
**

**D.**

**
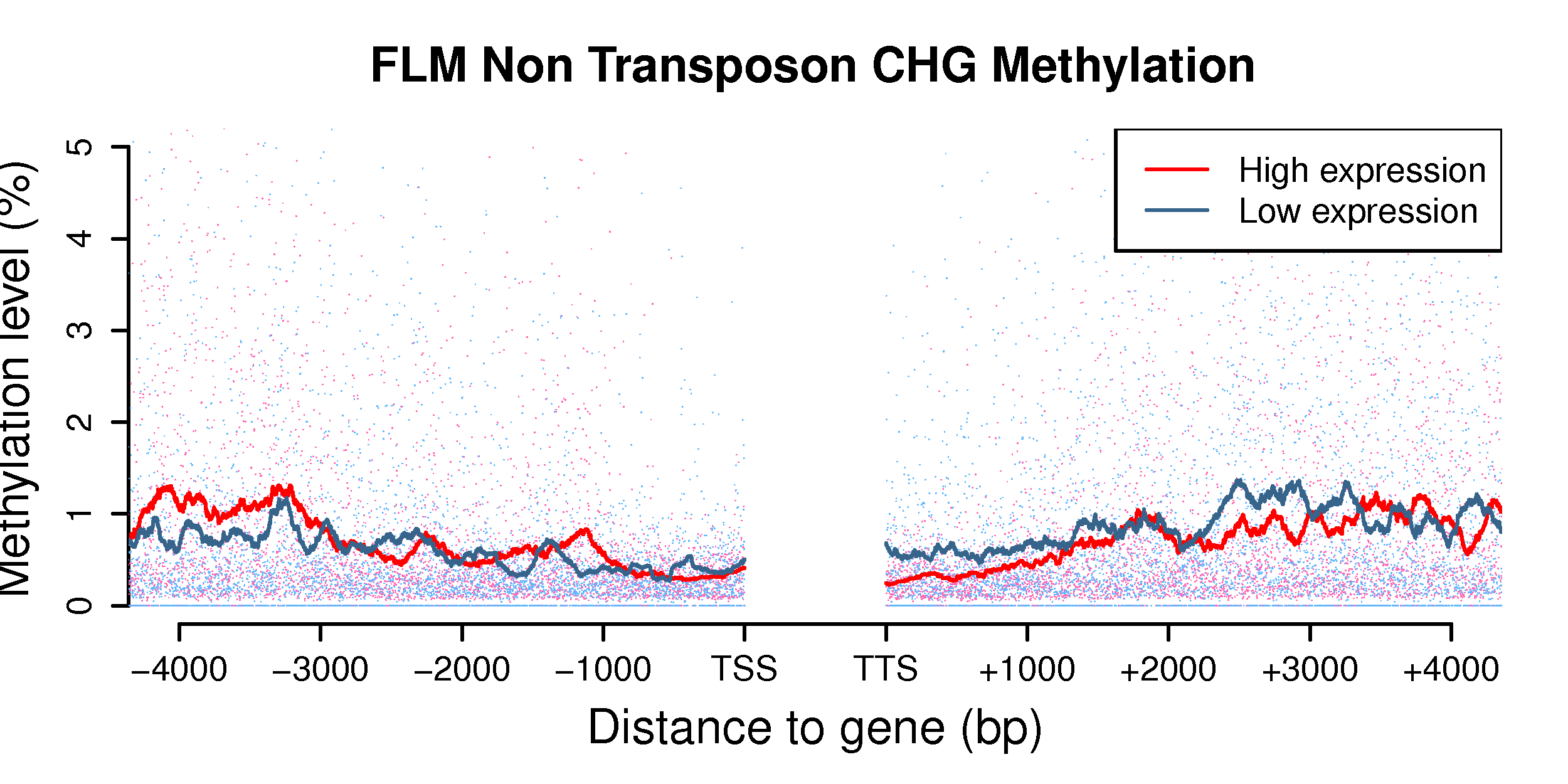
**

**Figure S11.** Non-transposon DNA methylation and neighbor gene expression levels for FB (CG sites), FB (CHG sites), FLM (CG sites), FLM (CHG sites) are shown in panels (**A**), (**B**), (**C**), and (**D**), respectively. Methylation levels are referred to non-TE DNA regions upstream or downstream to highly (top 25%) or lowly (bottom 25%) expressed genes as indicated. *Red* (highly expressed genes) and *blue* (lowly expressed genes) lines represent the moving average of methylation levels in 100 bp windows.


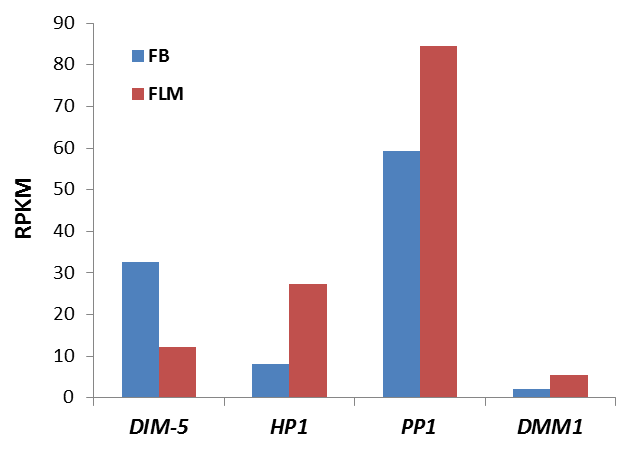


**Figure S12.** Expression levels of putative *T. melanosporum* DNA methylation accessory proteins in FB (*blue bars*) and FLM (*red bars*). Expression levels (RPKM) refer to the *T. melanosporum* homologs (gene IDs in brackets) of the following validated components of the *N. crassa* DNA methylation machinery: histone-lysine N-methyltransferase DIM-5 (GSTUMT00003241001); H3K9me3 histone binding protein, “heterochromatin protein 1” (GSTUMT00000912001); H3S10p phosphatase PP1 (GSTUMT00009673001); DNA methylation modifier DMM-1 (GSTUMT00000976001).


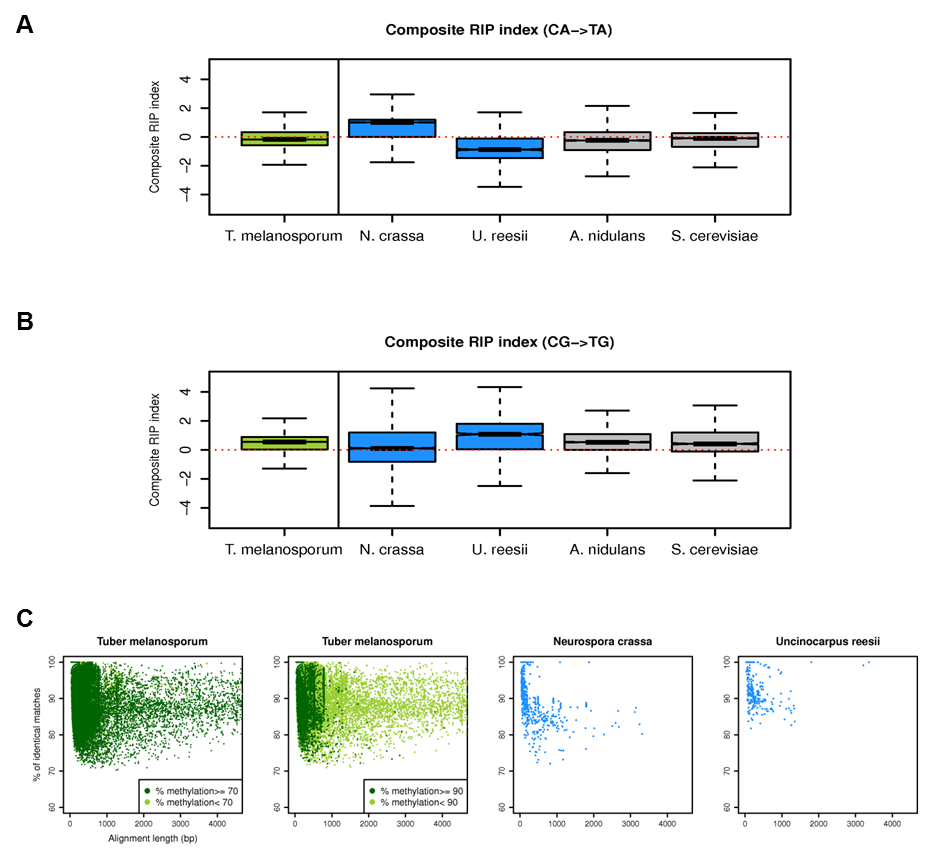


**Figure S13.** Cross-species genome comparisons between *T. melanosporum* and other fungi. (**A**) Box plots of the Composite RIP Index (CRI) for the truffle genome (*left panels*) and for the genomes of other fungi (*right panels*) based on CA to TA dinucleotide changes. (**B**) Same as (A) for CG to TG dinucleotide changes. Data were calculated as 50 kb genome-wide windows; deviation from baseline (CRI=0) increases with the likelihood of RIP occurrence. *N. crassa* served as a RIP positive control in panel (A); *U. reesii* was included in both analyses because of the exceptionally high CG->TG dinucleotide change frequency reported in a previous study [7]. (**C**) Scatter plots of sequence similarity versus alignment length in *T. melanosporum* are shown in the two *left-side panels*, where dark-green dots indicate methylation percentages ≥ 70% (*first left panel*) and ≥ 90% (*second left panel*) compared to *N. crassa* and *U. reesii* (*third and fourth* *panel*).


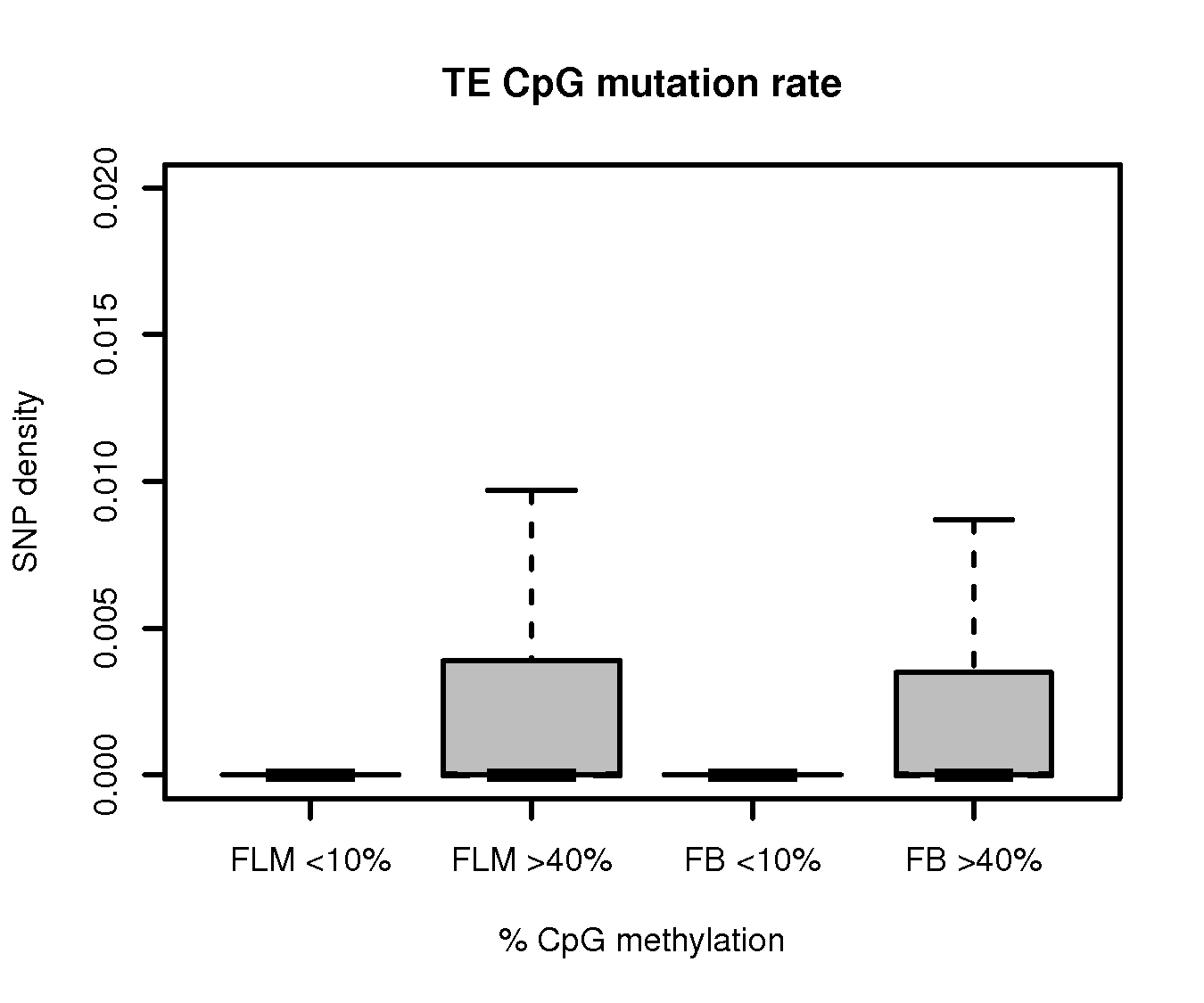


Low High Low High

FLM FB

CpG methylation

### Figure S14. Transposon CpG mutation rate as a function of DNA methylation. Mutations (single nucleotide polymorphisms, SNPs) on CpG sites were called from BS-seq data. For each TE, SNP density (i.e., mutation rate) was calculated. The box plot shows SNP density for highly (>40%) and lowly (<40%) methylated TEs; a high mutation rate appears to be associated with high TE methylation levels.
