# Supplementary figures and images for: Non-exhaustive DNA methylation-mediated transposon silencing in the black truffle genome, a complex fungal genome with massive repeat element content
Source: Genome Biol. 2014 Jul 31;15(8):411. doi: 10.1186/s13059-014-0411-5 (PMC4165359; doi:10.1186/s13059-014-0411-5)

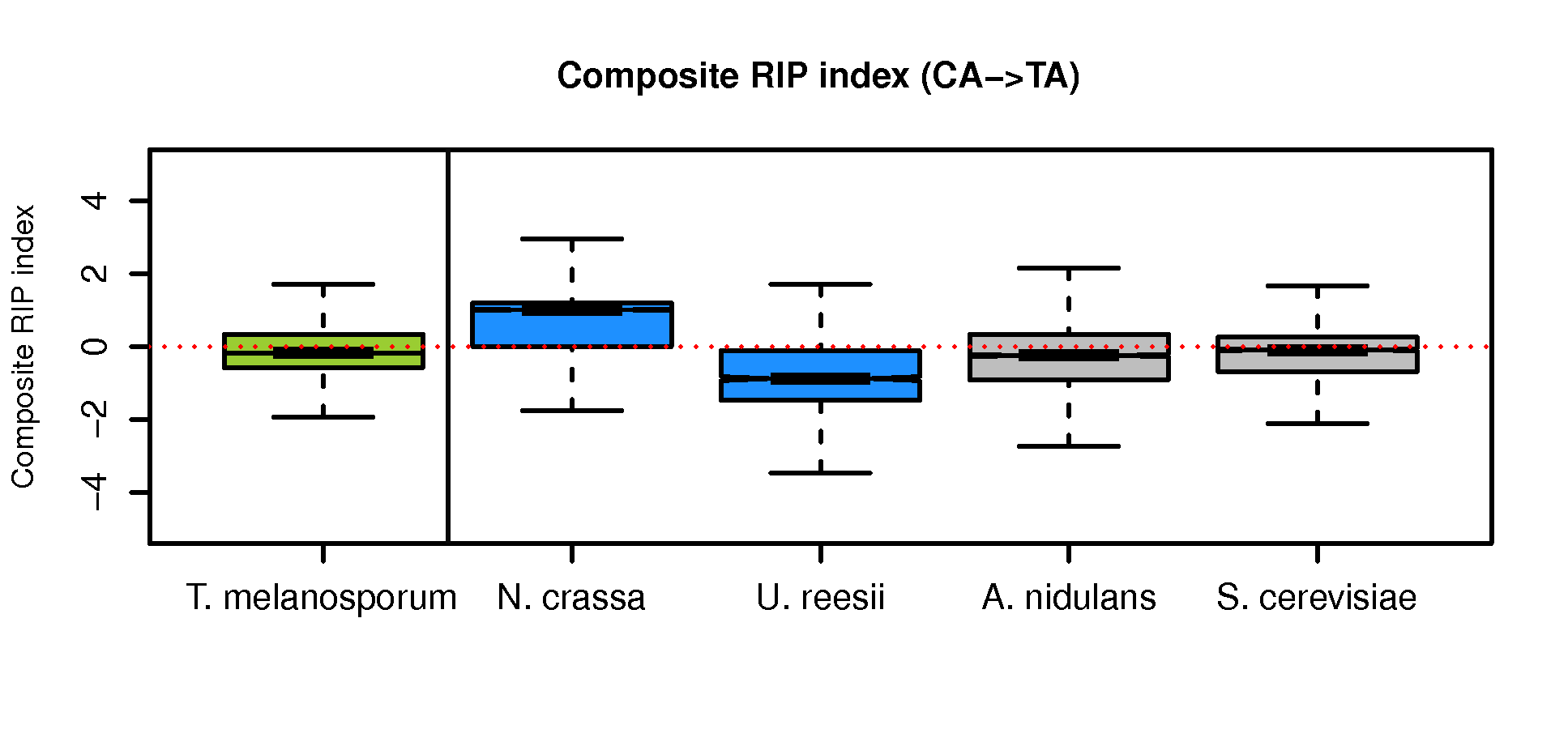

Supplement: Additional file 5 — The 10 output CRI files used to generate the plots shown in Figure S13A,B in Additional file 1 . [file 13059_2014_411_MOESM5_ESM.zip › Additional file 5/Box-CRI-repeats-CA-Tuber-vs-other4.png]

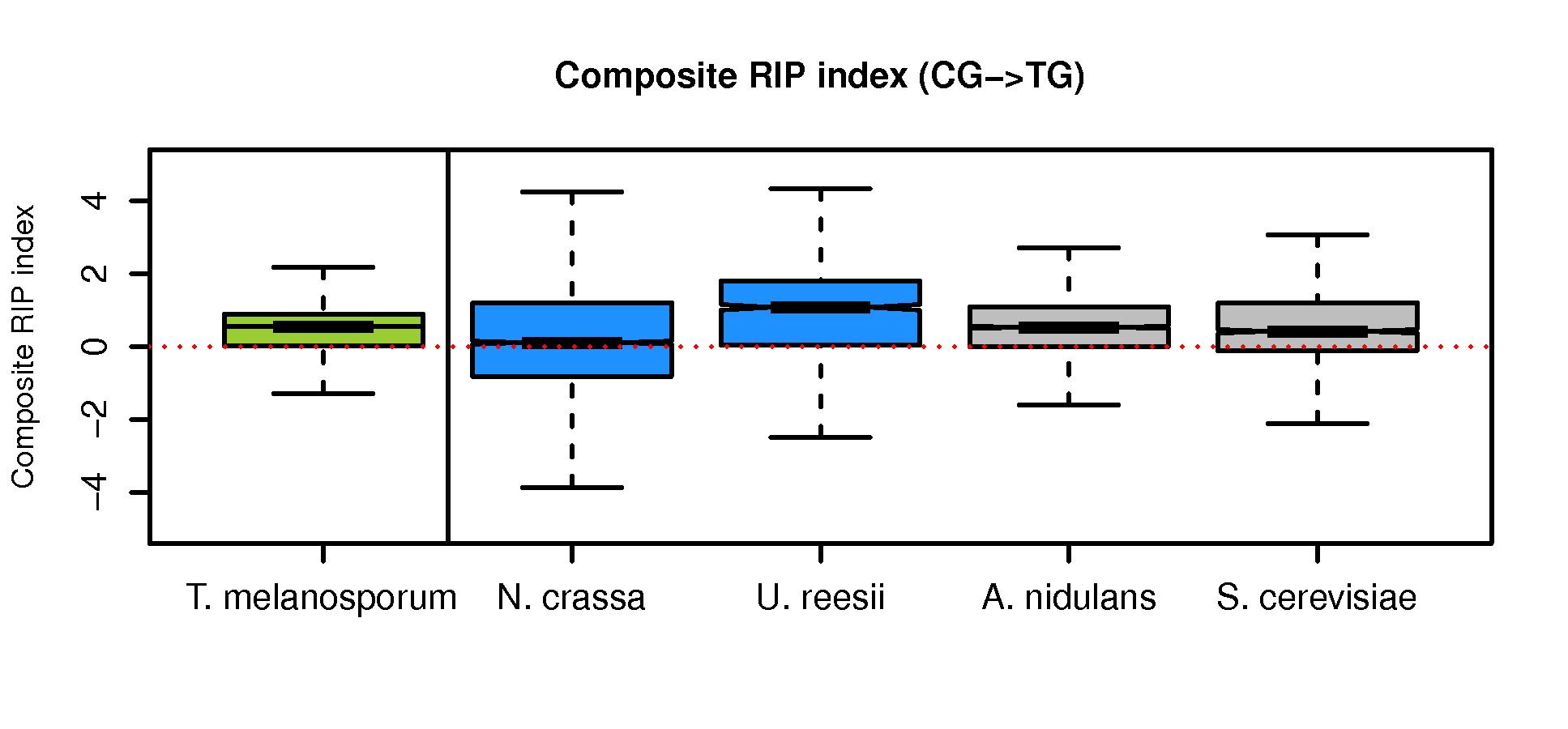

Supplement: Additional file 5 — The 10 output CRI files used to generate the plots shown in Figure S13A,B in Additional file 1 . [file 13059_2014_411_MOESM5_ESM.zip › Additional file 5/Box-CRI-repeats-CG-Tuber-vs-other4.png]
